# Antimyostatin Treatment in Health and Disease: The Story of Great Expectations and Limited Success

**DOI:** 10.3390/cells10030533

**Published:** 2021-03-03

**Authors:** Tue L. Nielsen, John Vissing, Thomas O. Krag

**Affiliations:** Copenhagen Neuromuscular Center, Department of Neurology, Rigshospitalet, University of Copenhagen, DK-2100 Copenhagen, Denmark; john.vissing@regionh.dk (J.V.); thomas.krag@regionh.dk (T.O.K.)

**Keywords:** myostatin, muscular dystrophy, muscular regeneration, ActRIIB, TGF-β

## Abstract

In the past 20 years, myostatin, a negative regulator of muscle mass, has attracted attention as a potential therapeutic target in muscular dystrophies and other conditions. Preclinical studies have shown potential for increasing muscular mass and ameliorating the pathological features of dystrophic muscle by the inhibition of myostatin in various ways. However, hardly any clinical trials have proven to translate the promising results from the animal models into patient populations. We present the background for myostatin regulation, clinical and preclinical results and discuss why translation from animal models to patients is difficult. Based on this, we put the clinical relevance of future antimyostatin treatment into perspective.

## 1. Introduction

Muscular dystrophies consist of a broad array of inherited conditions characterized by muscular wasting and atrophy. As clinical presentations in patients may vary due to a wide spectrum of phenotype–genotype variants for a particular gene, a common treatment, not depending on correcting a single molecular defect, has emerged as an attractive target for development. For the last 20 years, one of the most promising therapeutic subjects in the field of muscular dystrophies has been myostatin. Identified for the first time in 1997, myostatin knock-out in mice caused increased muscle mass [1] and mutations in the myostatin gene (*MSTN*) gene have subsequently been identified in the double muscled Belgian Blue and Piedmontese cattle [2,3,4] as well as whippet racing dogs [5]. In 2004, a loss-of-function mutation of *MSTN* in a German boy with a hypermuscular phenotype demonstrated that the effect of myostatin is functionally conserved across different mammalian species [6]. Since myostatin loss of function did not appear to have any negative impact on viability and longevity [7,8], interest was raised towards a novel treatment by harnessing the potential of inhibiting this negative regulator of muscular growth. Numerous studies in animal models and clinical trials have tried to explore this relationship with promising results in preclinical studies, which have translated poorly in human clinical studies. As the molecular and preclinical foundation for myostatin inhibition have been carefully reviewed before [9,10], this review will briefly describe the molecular involvement of myostatin in the muscle of humans and mice as well as healthy, diseased and exercising individuals. We will focus on the detailed results of the preclinical studies, the common denominators of these and we will present the results of the clinical trials in humans and how results in mice may or may not translate to humans. Finally, we offer perspective to a future path for myostatin inhibition with respect to the knowledge that the past 20 years of myostatin research has provided us with.

## 2. Molecular Involvement of Myostatin in Mice and Humans

**Figure 1:**
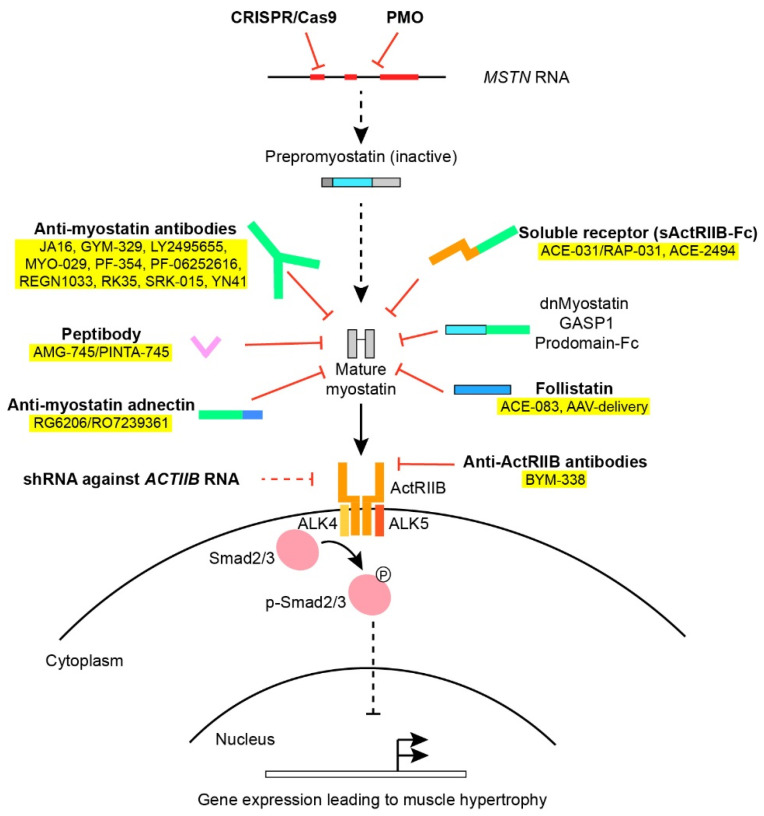
An overview of various approaches used in myostatin inhibition. Various factors and approaches in myostatin inhibition as outlined in Section 2 and Section 5. Treatments applied in clinical trials have been colored yellow. The Smad2/3 intracellular signaling pathway downstream the ActRIIB leads to altered gene transcription of muscle regulatory factors.

Myostatin, also known as growth and differentiation factor 8 (GDF-8), was identified in 1997 by McPherron and Lee [1]. During embryogenesis, myostatin is expressed in the developing epaxial and hypaxial myotomes [11,12] and hereafter in muscular tissue postnatally, but has also been found at low expression in adipose tissue, heart and circulation throughout development [13,14]. As the *mstn*-gene is highly conserved among different vertebrate species [3,6,15,16,17,18], it is evident that it has an important function in muscle development and physiology, which has been preserved during the course of evolution. As a member of the TGF-β-superfamily, myostatin shows homology to other growth and differentiation factors, such as bone morphogenic proteins (BMP) and activins, which also elicit their biological function as dimers. Prepro-myostatin is synthesized as an N-terminal signal peptide followed by a propeptide domain and eventually a mature C-terminal domain [15]. During proteolytic processing, the signaling peptide is removed and the propeptide is cleaved from the mature protein. As the mature C-terminal domain dimerizes and forms disulfide bridges, it remains inactivated since noncovalent bonds between the mature dimer and the propeptide hold the mature myostatin in an inactive state [13,19,20,21]. To exert its function, the propeptide must be cleaved from the inactive complex by a family of BMP1/TLD-metalloprotease proteins [21,22]. Other than the propeptide itself, regulation of myostatin activity is also known to be mediated by follistatin [19,23], follistatin-related gene (FLRG) [13], Gasp-1 [24] and the proteoglycan protein decorin [25,26] typically by blocking the binding of myostatin to the receptors (Figure 1).

Once liberated from inhibitory proteins, myostatin, as well as other members of the TGF-β-family, binds to activin-receptors and, in the case of myostatin, mainly to the activin-receptor type IIB (ActRIIB) as well as the type IA receptor [19]. The activin receptors are transmembrane serine/threonine kinases that subsequently recruit and activate dimers of type I-receptors (ALK4 and ALK5) [27,28]. Depending on the receptor ligand and the composition of the receptor complex, the type I-receptor will phosphorylate and activate intracellular protein Smad2 and 3 downstream to the membrane receptors through the canonical Smad-pathway. Smad2/3 binds to Smad4 and the complex translocates to the nucleus [29], where muscle regulatory factors MyoD, Myf5 and Myogenin are repressed [30], preventing myoblast proliferation [31] and differentiation [30]. Obstruction of the myostatin pathway inhibits activation of Smad2/3, making Smad4 available in the BMP signaling pathway which promotes hypertrophy and counteracts the effects produced by myostatin [32]. Other noncanonical pathways activated by myostatin involve (among others) AMP-activated protein kinase (AMPK) and p38 mitogen-activated protein kinase (MAPK) [33,34].

## 3. Myostatin in Healthy Humans and in Relation to Clinical Manifestations of Cachexia and Muscular Wasting

Compared to healthy young men, there was no reported change in serum myostatin levels in an elderly population with mild or severe sarcopenia (as defined by muscular contractile force) [35]. Burch et al. reported that myostatin was 57% higher in a healthy cohort >25 years of age compared to a healthy cohort <25 years, with an age-dependent increase in the younger cohort but not in the older cohort [36]. A different study with more than 1100 participating men aged 20–87 years demonstrated that circulating myostatin level was dependent on age and body mass index [37]. Additionally, men had higher levels than women [36]. This is in contrast to findings of myostatin levels declining on ageing in men both measured by ELISA [38] and immunoplexed liquid chromatography with tandem mass spectrometry [39]. A smaller study of eight young and six elderly women showed higher levels of myostatin mRNA in muscle biopsies of the older group [40]. Various groups have sought to determine the use of myostatin as a potential biomarker for muscle wasting but the conclusions have been ambiguous [41,42,43].

The effect of age on the expression of not only myostatin but also other promyogenic muscle regulatory factors (MRF) following exercise was examined by Raue et al. They found that at rest, there is a relative upregulation of both MRF and myostatin prior to exercise in elderly women compared to younger ones, but that the postexercise downregulation of myostatin is not hampered by age [40]. A study in healthy and sarcopenic elderly men demonstrated that resistance training or a combination of resistance and endurance training caused a decrease in myostatin [44,45].

The clinical relevance of myostatin in humans was described for the first time in HIV patients, who had increased levels of myostatin compared to healthy subjects. Furthermore, the levels were even higher in the patients who met the definition of AIDS-wasting syndrome [15]. The role of myostatin in muscular atrophy and muscle wasting was also determined in mice that developed cachexia in response to myostatin overexpression [46]. Cachexia manifests as a complex metabolic syndrome due to an underlying illness characterized by muscle wasting in conditions such as chronic heart failure (HF), cancer, chronic obstructive pulmonary disease (COPD) or chronic kidney disease (CKD) [47]. The use of myostatin inhibitors in such populations with progressive muscle wasting or atrophy secondary to an underlying condition is attractive, as the preservation of muscle strength for ambulation, personal care and everyday independence is key in reducing morbidity and improving quality of life.

In terms of cardiovascular disease, the upregulation of myostatin in the cardiomyocytes surrounding an ischemic infarction in sheep was shown in 1999 [14] and myostatin protein and mRNA in skeletal muscle and myocardium were increased in a rat-model of volume overload heart failure [48]. Lenk and colleagues also found that the protein expression of myostatin was increased in the skeletal muscle and myocardium of a murine LAD-ligation heart failure model, which corresponded to later findings in chronic heart failure patients who had elevated levels of myostatin mRNA and protein in muscle biopsies compared to healthy controls [49,50]. The relationship between myostatin levels in the circulatory system and patients suffering from chronic heart failure has been examined by various groups. Increased myostatin levels in HF-patients could be expected, since impaired cardiac output reduces oxygen supply to the vascular bed of muscle tissue and less muscle means less oxygen consumption. As various studies have detected elevated [50,51,52], equal [53] or lower [42,54] levels of the latent and inactivated myostatin complex in the circulatory system, methodological differences in the detection of myostatin (e.g., Western blotting of promyostatin versus immunoassays of full-length myostatin and Liquid chromatography–mass spectrometry (LC–MS)) may account for these fluctuating results [36]. Furthermore, myostatin levels in decompensated chronic HF patients dropped upon compensation therapy, suggesting dynamics and variability in myostatin levels, which are sensitive to therapeutic interventions [55].

Treating cancer-associated cachexia by means of myostatin inhibition has been another field of interest. As myostatin was elevated in the gastrocnemius muscle of mice inoculated with the Yoshida AH-130 hepatoma [56], targeting the myostatin pathway seemed promising in preventing cancer cachexia. C26-tumor-bearing mice were treated with a soluble receptor of the ActRIIB (sActRIIB), which improved survival and muscle mass without reducing tumor size [57] and by treating the Lewis lung cancer-model with myostatin antibodies, muscular atrophy and loss of muscle force were attenuated [58].

COPD has been another target of interest due to the muscle wasting, since 30–40% of all people with COPD undergo muscle wasting as a secondary complication to impaired pulmonary function [10]. The link between myostatin and chronic hypoxemia was established in rats exposed to chronic hypoxia, which induced myostatin expression in rat muscle [59], and the increased the expression of myostatin in the vastus lateralis and serum of COPD-patients compared to healthy controls has also been described [59,60]. Later, serum myostatin was found to be significantly elevated in COPD-patients compared to controls but skeletal muscle mass only correlated negatively with serum-myostatin in males [61].

In CKD, myostatin is elevated in the serum and skeletal muscle of the rat model of CKD, (*Cy/+*), with increased activation of atrogenic transcription factors in EDL adding insights to the pathophysiology behind muscle wasting in this condition [62].

## 4. Myostatin in Response to Exercise

The effect of exercise on the expression of myostatin has been demonstrated numerous times. In a clinical study where subjects had immobilized a limb for two weeks following exercise rehabilitation, the casting-induced atrophy did not affect myostatin mRNA in muscle biopsies. However, exercise led to downregulated myostatin expression by approximately 48% [63]. These findings indicate that myostatin works in vivo by inhibiting hypertrophy, rather than inducing atrophy. Similar findings in exercise studies have been observed up to 24 h after exercise [64,65] and on protein-level in prediabetic patients performing moderate aerobic exercise for six months [66]. Most interestingly, “the myostatin paradox” was introduced by Kim et al., who in their exercise study discovered a positive correlation between myostatin mRNA and muscle mass [64], whereas the relationship would most intuitively be the opposite if not taking inhibitory factors into consideration. The authors speculate that high levels of myostatin transcripts in muscle might prime the muscles for additional growth.

## 5. Preclinical Studies of Myostatin Inhibition in Animal Models of Neuromuscular Disorders

The potential for the pharmacological regulation of muscular growth had to be explored in animal models of muscular dystrophy, atrophy and muscular regeneration before ultimately turning towards clinical trials in human subjects. We present here an overview of the various ways in which myostatin has been targeted in animal models. As myostatin inhibition has been utilized to examine various physiological processes other than merely muscular regeneration (including cancer survival, bone- and energy metabolism), the following will focus on the bulk of scientific work that describes the effect of myostatin on muscular tissue. A summarized overview is presented in Table 1 with the detailed results of the single publications available in Supplementary Table S1. Table 1 presents information, if available, on the animal model and genus, the pharmacological compound, muscle morphology, fiber-type specific changes, absolute and specific force amongst glycolytic and oxidative muscles, muscular stress resistance and histopathological improvements. This review is focused in particular on treatment-mediated functional improvements of muscle function, as these are essential for any translation to human clinical trials. Histopathological recovery, muscular growth and the upregulation of desirable growth factors or genes in vitro may be of less importance, as primary outcomes are invariably functional in preclinical studies and the degree of functional improvement ultimately decides whether a treatment will advance in additional preclinical or clinical investigations. Furthermore, increasing the absolute force is of interest to patients and clinicians who are looking for improvements in the activities of daily living, while the scientist will be looking for specific force (force per cross sectional area of a muscle) as an indicator of whether the underlying deficit has been compensated for.

The pharmacological approaches to inhibiting myostatin activity in vivo have included: (a) systemic administration of antibodies against myostatin; (b) overexpression or administration of the myostatin propeptide; (c) systemic administration of the activin-IIB-receptor itself; (d) administration of antibodies directed against ActRIIB; (e) overexpression or administration of follistatin; (f) liver-mediated overexpression of a soluble receptor (sActRIIB), dominant-negative myostatin (dnMSTN) or the propeptide; (g) RNA interference and antioligonucleotides against myostatin or ActRIIB or; (h) AAV-Cas9 mediated myostatin gene editing. Finally, we have also included works on the effects of transgenic knock-out models and crossbreeding with the preexisting models of muscular dystrophy (i).

### 5.1. Antibodies against Myostatin

Bogdanovich et al. were the first to successfully treat the commonly used mouse model of Duchenne Muscular Dystrophy (DMD), the *mdx*, with antibodies directed towards myostatin (monoclonal antibodies, JA16) [73]. The results were promising, as the diaphragm and the skeletal muscle, which in the *mdx* reproduces the pathological features seen in muscles of DMD-patients most accurately [132], showed fewer degenerative features compared to controls. Meanwhile, m. extensor digitorum longus (EDL) had increased weight, cross-sectional area (CSA) and absolute force but failed to show improvement on specific force and stretch resistance. Similar results with increased muscle weight and absolute force but lack of improvement in specific force and resistance were seen in the *Sgcg^−/−^* model of limb-girdle muscular dystrophy (LGMD) 2C in a design of similar age and treatment length to the previously mentioned study [74]. The *Sgcd^−/−^* mouse model of LGMD2F also treated with JA16 antibodies was not able to improve fibrosis in either young or older *Sgcd^−/−^* animals (4 and 20 weeks old at treatment start, respectively) with older animals even showing signs of worsening of fibrosis [75]. Interestingly, a 5-week treatment period of very young (16 days old) *mdx*-animals showed positive effects on the diaphragm, as specific force increased while absolute force was unaffected, fiber size increased and connective tissue infiltration of the diaphragm was reduced [76], indicating that early initiation of treatment is crucial for a positive effect. Another monoclonal antibody developed by Pfizer, mRK-35, was also able to increase absolute but not specific force in *mdx* mice [72] and the Tg*Acta1*^D286G^ mouse model of nemaline myopathy [78]. Treatment of the *Sod1*^G93A^ mouse and rat models of amyotrophic lateral sclerosis (ALS) with RK35 improved grip strength compared to placebo controls but did not delay disease onset or extend survival of either model [80]. Later, Muramatsu and colleagues introduced the concept of “sweeping antibody technology” with the GYM329-antibody designed to bind and clear latent myostatin from the circulatory system, which increased muscle mass in three mice models and cynomolgus monkeys and also improved grip strength in the mice [71]. As opposed to other antimyostatin antibodies, GYM329 did not bind GDF-11 and this specificity appears to induce an enhanced effect on muscle mass in treated animals. Especially in older animals, where other myostatin inhibition treatments fail or struggle to achieve an effect, GYM329 appeared superior. Other models of neuromuscular disorders such as the *SmnΔ7* mouse of spinal muscle atrophy (SMA) had increased absolute muscle torque but not specific torque after treatment with the muSRK-015P antibody versus myostatin (combined with salvation of *Smn*2-gene mRNA) [133]. In a study of micro-gravity-induced muscular atrophy, mice were held at the International Space Station and treated with YN41 for 6 weeks, inducing improved grip strength compared to controls, as well as increased muscle mass [68].

### 5.2. Myostatin Propeptide Administration or Overexpression

As previously mentioned, the myostatin propeptide functions as an inhibitor of myostatin, as it binds myostatin in an inactive complex. Propeptide-based inhibition by intraperitoneal injection for three months resulted in increased body mass, EDL mass, absolute and specific force in EDL. There was no effect on stretch-resistance but the histopathological phenotype of the diaphragm improved compared to untreated *mdx* [84]. Bartoli et al. treated calpain-3-null and *Sgca*^−/−^-mouse models of LGMD2A and 2D, respectively, by local and systemic overexpression of the propeptide but were only able to improve the calpain-3-null mice [86].

### 5.3. Systemic Administration of the Soluble Receptor ActRIIB

In order to increase the specific targeting of myostatin and reduce binding of the variety of other ligands that also bind to and activate ActRIIB, another approach based on the systemic administration of a soluble activin type IIB receptor, sActRIIB, has been widely utilized (Supplementary Table S1). The compound RAP-031, developed by Acceleron, is a fusion protein consisting of the extracellular domain of the ActRIIB linked to the Fc-portion of murine IgG to delay systemic clearance. Applying this approach, Pistilli and colleagues demonstrated an increase in both absolute and specific force of EDL in *mdx* mice [98], a functional outcome, which unfortunately has been difficult to replicate in both wild-type [87,93], *mdx* [92,94,99], and nemaline myopathy mouse models [101] (Supplementary Table S1). The treatment of mice with muscular atrophy due to spinal cord injury with RAP-031 did not alleviate the atrophy [134]. A hypoxia model in wild-type mice showed improved resistance to eccentric lengthening but no other studies using the soluble receptor have shown improvements to stretch resistance [135]. The specific hypertrophy of fibers with a IIB fiber-type composition was observed in two models of myotubularin-deficient mice [90,102] but also in other fibers of wild-type animals [89,90].

### 5.4. Administration of Antibodies Directed against ActRIIB

Blocking the ActRIIB itself by antibodies has not been widely used as another means of myostatin inhibition. Novartis developed BYM338 (bimagrumab, which would progress into clinical trials as mentioned below) and described the receptor-specificity in cell cultures and myoblasts while also showing the effects on body and muscle mass in both SCID-mice and a glucocorticoid atrophy model [106].

### 5.5. Follistatin Administration or Overexpression

Like the myostatin propeptide, follistatin is able to inhibit not only myostatin but also shows affinity for other TGF-β-family members (such as BMPs and activins) [24,136]. Transgenic overexpression of follistatin primarily showed increased muscle weight and fiber diameter [19]. Transgenic mice overexpressing a follistatin-derived myostatin inhibitor crossed with the *mdx* ameliorated the dystrophic features in terms of grip strength and pathohistological features [137]. When transgenic overexpression of follistatin (*F66*-mice) is crossed with the dysferlinopathy LGMD2B model *Dysf*^−/−^, the positive effect on muscle weight in *F66;Dysf^−/−^*-mice declines with age and the specific force of EDL is reduced, compared to *F66*-mice, exacerbating the dystrophic phenotype [104]. Furthermore, ActRIIB-FC-administration in *Dysf*^−/−^-mice ameliorated histopathological changes, but increased creatine kinase (CK, a marker of muscular damage and membrane integrity) levels. The authors conclude that follistatin overexpression accelerated the degenerative features in the dysferlinopathy model, as the dystrophin-deficient *mdx* was not exacerbated [104], and suggest that muscle hypertrophy may have pernicious effects depending on the disease context.

Another approach using a follistatin-based fusion protein ACE-083 by local intramuscular injections increased CSA, weight and absolute, but not specific, force of injected muscle tibialis anterior (TA) in the Trembler-J mouse model of Charcot–Marie–Tooth disease and *mdx* [111].

As the systemic clearance of follistatin is rather quick, systemic versus local administration poses a challenge. Thus, the pharmacokinetic properties were edited and a long-acting follistatin-based molecule (FS-EEE-hFc) was engineered by Shen and colleagues [138] and applied by intravenous and subcutaneous administration to wild-type and *mdx*-animals [99]. The subcutaneous treatment of young (4 weeks) *mdx*-mice for 12 weeks also undergoing an exhaustion-exercise regime showed increased muscle weight and absolute but not specific force increments [99].

### 5.6. Liver-Mediated Overexpression of Dominant-Negative Myostatin (dnMSTN), sActRIIB and Myostatin Propeptide

Using the same approach as mentioned earlier with adeno-associated virus 8 (AAV8)-delivered myostatin inhibitors, Morine and colleagues treated the *mdx* with AAV-vectors, which brought liver-mediated transcripts of sActRIIB [115] or dnMSTN [116] into circulation. The sActRIIB treatment did increase the muscle mass, fiber size and absolute force of the EDL, while CK decreased. However, there were no positive effects in soleus or specific force [115]. The dnMSTN paper showed that the treatment in *mdx*-mice was predominantly observed in the fast fibers (IIA, IIX and IIB) of both the EDL and soleus, while soleus increased both absolute and specific force and CK decreased [116]. A similar study in the *D2.mdx* only reported beneficial effects on absolute force in EDL [117].

Similar to the treatment regimes in the *mdx*, Liu and colleagues treated the *C/C* mouse model of spinal muscle atrophy (SMA) with AAV8-vectors containing transcripts for dnMSTN and sActRIIB, respectively [114]. Both treatments increased the size of type IIA and IIB-fibers, leaving type I-fibers unaffected (IIX was not measured). While specific force was unaffected by treatment, absolute force increased in EDL (both treatments) and soleus (only sActRIIB).

Another approach was used in wild-type MF-1 mice, where propeptide coupled to an immunoglobulin Fc molecule was delivered by means of AAV8 vectors to hepatocytes, ensuring an intrinsic production of the inhibitor [113]. In contrast to exogenic injections of the propeptide, Foster and colleagues treated mice from six weeks of age and found an increased absolute force in oxidative muscle soleus but not in EDL. Both EDL and soleus increased CSA, as well as subanalyses of fiber-types I, IIA and IIB. In a similar design, *mdx*-mice were treated at the age of three months, which increased body mass, grip strength, muscle mass and fiber radius [85]. The absolute twitch and tetanic force production improved but specific force did not.

### 5.7. RNA Interference and Antioligonucleotides against Myostatin or ActRIIB

Myostatin has also been sought downregulated by means of RNA interference. Dumonceaux et al. combined short hairpin RNA (shRNA) interference of ActRIIB mRNA with AAV mediated exon-skipping of dystrophin. The number of fibers increased in TA, but force production was unchanged in mice that received myostatin interference solely compared to untreated *mdx* [120].

In contrast to AAV-mediated gene therapy, antisense oligomers (AOs) hold no risk of uncontrolled genome insertion and levels of exon skipping can be regulated or aborted over time. Antisense phosphorodiamidate morpholine oligomers (PMOs) causing exon-skipping of myostatin increased TA weight and CSA locally in *mdx*-mice [139]. A follow up study combining systemic treatment with two different PMOs that restored dystrophin and inhibited myostatin, respectively, was promising but the *mdx* mice receiving the myostatin-inhibiting PMO did not benefit from this treatment alone [119]. A similar study demonstrated similar increases in muscle mass in PMO-skipped myostatin, but also demonstrated that skipping varied among muscles, with the highest level of skipping in the soleus. These studies emphasize the importance of the design of the PMO, as well as the variable results obtained in healthy and *mdx* animals, suggesting that histopathology plays a role in efficiency of the treatment [119,139].

### 5.8. AAV-Cas9-Mediated Myostatin Gene Knock-Down

Recently, it was demonstrated that myostatin knock-out by the means of AAV-Sa-Cas9 gene editing delivered by intramuscular injections increased fiber area and number of fibers per area in aged wild-type mice [79]. However, functional outcomes were not described.

### 5.9. Crossbreeding Transgenic Myostatin Knock-Out Animals

The murine hypermuscular myostatin knockout (*Mstn*^−/−^, also denominated ‘the myostatin-null’) described in 1997, has subsequently been further examined and crossed with various mouse models of neuromuscular diseases. The myostatin-null itself has been described numerous times [1,123,127] with increased muscle and body mass. Force measurements have shown both positive and no effect on absolute force in the myostatin knock-outs but decreased specific force has generally been reported [121,122,123] (Supplementary Table S1). An increased proportion of fast fiber-types has been the common observation [123,124,125,126,127], in line with the findings in studies of pharmacological myostatin inhibition (see above). Another model of myostatin malfunction includes the Compact-mouse (also known as the Berlin High Line BEH^C/C^), which contains a 12-bp deletion in the propeptide domain of promyostatin (*Mstn*^Cmpt-dl1Abc^) but leaves the biologically active growth-factor domain of myostatin unaffected [140]. Kocsis and colleagues later found that the Compact genetic background itself, in addition to the promyostatin genetic deletion, determines the phenotype [128] and the use of this model has been rather limited.

A third mouse model is the lean myostatin mouse (*Mstn*^ln/ln^), which has an induced loss-of-function mutation leading to a peptide without the ligand, thus a complete lack of myostatin. This model has similarly increased muscularity but has had most of its use in the field of metabolic research [141].

Crossing myostatin-null with other models of muscular dystrophy has occasionally been the preceding study to pharmacological interventions. Crossing myostatin-null with *mdx* [129,130] or caveolin-3-deficient mice with transgenic mice overexpressing the myostatin prodomain (“MSTNPro”) [105] did ameliorate the pathological features by increasing body weight, fiber numbers and improving grip strength. However, the crossing of myostatin-null mice with the *dy*^W^/*dy*^W^ laminin-deficient mouse model of congenital muscular dystrophy failed to improve the dystrophic phenotype and postnatal lethality was even increased [131].

In addition, a recent study crossing a follistatin overexpressing mouse strain with the calpain 3 knock-out mouse model for LGMD2A led to increased glycolytic muscle mass, but caused the loss of AMP-activated protein kinase signaling, important for contraction-induced glycolysis and poor exercise tolerance [142].

## 6. Common Denominators in Animal Studies

It is evident that myostatin holds the potential for increasing hind limb muscle mass almost regardless of which muscles are investigated (Table 1). The increases in mass most likely reflect fiber hypertrophy and increased CSA, rather than hyperplasia, with the effect specific to fast glycolytic fibers. This is supported by evidence in myostatin-null mice where fiber-type switch from oxidative (“slow”) fibers towards glycolytic (‘fast’) is seen (Supplementary Table S1). A shift towards a more glycolytic fiber-type in animals treated with inhibitors has been reported [78,113,114,116] as well as a decrease in glycolytic fibers [82,91,92,101,119,124,143,144]. These different observations in fiber type changes make it difficult to establish a consensus on the overall effect of myostatin inhibition. EDL has been shown to have a higher expression of the ActRIIB than soleus [115,121] and as IIB fibers are associated with the highest content of myostatin [145], this could explain a differentiated effect favoring glycolytic muscle. Soleus, on the other hand, contains a fiber-type composition, which resembles a human muscle more closely (58% type I-fibers in the *mdx* [146] and 70% in wild-type mice [124]). The role of fiber-type differences in hind-limb muscle has not yet been resolved but unknown confounding factors may lie in the muscle of choice. Due to the overwhelming content of glycolytic muscle in the mouse [147], myostatin inhibition studies are almost guaranteed a positive effect on mouse muscle mass, as evidenced from many publications.

Looking at the histopathological changes, both qualitative and quantitative measures have been made when assessing the effect of treatment on fibrosis by visualizing and measuring e.g., hydroxyproline or collagen content. As with the functional studies, histopathological examination has shown both positive and negative findings (Table 1) while increased fiber diameter is a general finding. In terms of the CK levels, our examination of the literature shows that the ratio of successful to unsuccessful findings is moderately better but definitely not all studies are able to decrease CK in dystrophic animals.

On a functional level, most studies of postnatal myostatin inhibition present increases in absolute force, but very few studies [76,84,98,116] have been shown to increase specific force. Like specific force, resistance to stretch in eccentric contractions is a hallmark of translatable improvement in muscle function and noticeably myostatin-null mice have also shown a decreased specific force production in EDL due to fragile tendons [123,148]. Resistance to stress is a particularly difficult outcome to improve, mostly because the inhibition of myostatin does not remediate the original problem, which in the majority of the models is a compromised sarcolemma (Table 1). On the contrary, increased muscle mass will increase the stress on the sarcolemma and the treatment may compromise the tendons, as is seen in the *Mstn*^−/−^ mice. Not all dystrophic models have benefitted from myostatin inhibition; indeed, in those disease models where the sarcolemma or extracellular matrix is specifically affected, the treatment may cause further deterioration. The overexpression of follistatin in the dysferlin deficient *Dysf*^−/−^ mouse resulted in the exacerbation of muscular degeneration [104], older δ-sarcoglycan-deficient *Sgcd*^−/−^ mice treated with myostatin inhibitors showed signs of increased fibrosis [75] and the crossing of myostatin null-mice with laminin α2-deficient *dy*^W^/*dy*^W^ mice caused increased mortality in offspring [131]. The overall picture show that the fiber hypertrophy, which is seen in the most preclinical studies of myostatin inhibition, may not always be beneficial since small fibers are shown to have a lesser susceptibility towards necrosis [149]. We speculate that the fiber hypertrophy adds greater stress load on the single fiber which, in case of dystrophic muscle, has less endurance to withstand such force compared to a nondystrophic muscle fiber. Therefore, an increase in muscle mass can be fatal to the fiber if the membrane-associated proteins are not reinforced as well [150]. In continuation hereof, myostatin inhibition may show more promising results in a setting where a pathological loss of muscle mass is not complicated by inherited or acquired metabolic, immunological or mechanistic malfunction.

From a patient perspective, these limitations to the treatment mean that a large part of human muscle is either not responsive to the effect of myostatin inhibition or only to a minor degree as humans do not express type IIB fibers. Importantly, myostatin inhibition based on the mouse studies is unlikely to have any meaningful effect on the heart to halt or reverse cardiomyopathy and the degeneration of the muscles involved in respiratory function, as these are composed of oxidative fibers [151,152] (human heart and diaphragm) or type IIA-fibers [153] (murine diaphragm).

## 7. Clinical Trials in Myostatin Inhibition

Clinical trials using myostatin inhibitors have covered both DMD, the milder phenotype Becker Muscular Dystrophy (BMD) and LGMD, idiopathic inflammatory myopathies (sporadic inclusion body myositis, sIBM), cancer patients, COPD and a geriatric patient population (sarcopenia and weak fallers) (Table 2 and detailed overview in Supplementary Table S2).

### 7.1. Clinical Trials in Muscular Dystrophy

The first study in a population of muscular dystrophy patients (DMD, BMD, LGMD) receiving myostatin inhibitors was a phase I/II trial with MYO-029 (stamulumab, Wyeth Pharmaceuticals, now Pfizer) [154]. The clinical trial was designed for tolerability and adverse effects, which were quite few and limited to hypersensitive skin reactions in cohorts receiving high doses, but the biological and functional effects were nondetectable, attributed to a heterogenic study population and limited statistical power. Subsequent pharmacokinetic and -dynamic measurements suggested that the concentration of MYO-029 required to evoke a 50% improvement in monkey muscle was approximately 20x higher compared to an equivalent response in mice, indicating a significant potency-shift among species [83]. Later, the antibody PF-06252616 (domagrozumab, Pfizer), which neutralizes myostatin by binding to the mature myostatin dimer, increased lean body mass (LBM) and muscle volume by 5 and 4% in healthy subjects [155] but was unable to show an effect in DMD patients, and the phase II trial was terminated prematurely [156].

A phase I study of ACE-031 (ramatercept, Acceleron), a fusion protein of ActRIIB to the Fc-portion of human IgG, showed increased LBM in healthy women [161] but was unsuccessful in showing any effects when administered to DMD patients in a phase II trial and was retracted by the sponsor [163]. Adverse effects such as telangiectasias and epistaxis were reported and attributed to the binding of ActRIIB to other ligands, such as BMP9 and BMP10, involved in angiogenesis. Acceleron also developed ACE-083, a modified form of follistatin linked to human immunoglobulin Fc-portion, engineered to trap members of the TGF-β-family locally when injected into the muscle. Muscle volume increased in healthy subjects but failed to improve strength [165] and was unable to reach secondary end-points in following phase II trials including Charcot–Marie–Tooth (CMT) and facioscapulohumoral muscular dystrophy (FSH) (Table 2).

A different molecular strategy was pursued by Roche/Greentech with the antimyostatin adnectin RG6206/RO7239361, an engineered molecule based on a fibronectin III domain, which like antibodies binds a target molecule with high affinity. A phase Ib/II study reported increased LBM in treated DMD boys and the compound was found safe and well tolerated [166], which sparked a IIb/III study in which results have not been reported, but the phase II/III study was discontinued [167].

As a treatment for inflammatory myopathies such as spontaneous inclusion body myositis (sIBM), phase II and III trials of BYM-338 (bimagrumab, Norvartis), an anti-ActRIIB-antibody in sIBM failed to show positive long-term functional effects [168,169,170].

In a small phase I/IIa study of six BMD-patients without controls or placebo treatment, local injections of AAV1-delivered vectors harboring follistatin showed no adverse effects [175]. A subsequent phase I/IIa study of similar size combining the follistatin gene therapy, including an exercise regime in sIBM-patients compared to a control group, showed increased 6MWT distance and improved histopathological changes on muscle biopsy [176].

### 7.2. Clinical Trials of Other Applications of Myostatin Inhibition

As inherited muscular dystrophies such as DMD represent the most severe and irreversible conditions (here ignoring potential gene restoration therapies), it was considered that myostatin inhibition would show more promising results in milder phenotypes of the muscular disorder and conditions not arising from specific and inherited genetic malfunction. It can be speculated that the physiological adaptations in conditions arising from congenital genetic defects may be much harder to overcome by myostatin inhibition compared to acquired and potentially reversible conditions.

LY2495655 (landogrozumab, Lilly), a myostatin antibody, was tested in a population of elderly subjects who had experienced falling at least once before enrollment in the trial. Functional outcomes such as stair climbing time, chair rise with arms and fast gait speed increased, although the increase was not significant from placebo in all measures [159]. LY2495655 has also been tested in muscle wasting conditions associated with COPD and advanced pancreatic cancer, which are both conditions characterized by the loss of muscle, decreased physical function and overall performance status; measurements that are often crucial when determining eligibility for certain treatment regimes, such as anticancer therapy. LY2495655 failed to improve overall survival in the pancreatic cancer trial [158] and even though muscle mass improved in the COPD study, functional improvement was also absent [174].

BYM-338 was also examined in an older population suffering from sarcopenia with reduced gait-speed and muscle function. A single infusion of 30 mg/kg confirms a positive functional effect, but only when measuring the 6-min walk test (6MWT) distance 16 weeks after treatment in a subpopulation with low 6MWT performance at baseline [173]. Later, a monthly dose of 700 mg bimagrumab versus placebo for 6 months in combination with personalized exercise programs, dietary counseling and oral nutritional supplements did not show any effect of bimagrumab [171]. In a COPD-population, two doses of BYM-338 over 24 weeks were, as in the LY2495655-study, able to increase muscle mass but not functional outcomes [174]. However, casting-induced muscle atrophy in healthy men was reversed and recovery was accelerated when treated with a single dose of intravenous BYM-338 compared to placebo [173].

The antimyostatin peptibody AMG-745, developed by Amgen, is a fusion protein with a human Fc at the N-terminus and a myostatin-neutralizing peptide at the C-terminus [177]. Subcutaneous administration for four weeks increased muscle mass at follow-up one month after the final dose but functional improvement and strength did not improve in this study. In conclusion, myostatin inhibition in nondystrophic subjects can improve muscle mass but the functional improvement is highly questionable, even in these heterogeneous populations.

## 8. The Lack of Effect of Myostatin Inhibition in Clinical Trials of Muscular Dystrophy

Evidently, not a single clinical trial in muscular dystrophy has succeeded in reaching a clinically significant outcome and most have been withdrawn (Table 2). Why is this? A possible explanation could be decreased myostatin levels in DMD-patients, which have been reported to be approximately 70% lower compared to healthy age-matched controls and that there is a significant decrease in myostatin with ageing and loss of ambulation in DMD patients—suggesting that disease progression plays a role in circulating myostatin levels [36]. Second, circulating myostatin is at least 20-fold lower in humans compared to mice, making human muscle a poorer target for myostatin inhibition than what preclinical results would suggest [36], in continuation with the pharmacodynamic differences also mentioned in relation to the MYO-029 clinical trial [83]. Third, the downregulation of the myostatin pathway downstream of the receptor in atrophying or wasting muscle has also been suggested as an explanation [178]. Fourth, as generally all DMD patients have been treated with corticosteroids, the role of prednisolone in myostatin inhibition was examined in both the *mdx* and the more severe D2.*mdx* [117]. Hammers and colleagues demonstrated that not only does prednisolone induce skeletal atrophy, but the overexpression of myostatin cannot rescue such iatrogenic muscle wasting. Fifth, a recent treatment study with the GYM329 and three competitive myostatin antibodies implies that specificity against myostatin matters [71]. Since the ActRIIB binds several ligands involved in growth control and bone formation, blocking this may affect more than just myostatin [32]. In addition, some myostatin antibodies also bind GDF-11, which has been demonstrated to lead to a cap on the effect on myostatin. The unintended effects of these side-effects may hamper the true potential of inhibiting myostatin to increase muscle mass. Sixth, as type II fibers are the first to degenerate and eventually become lost in DMD patients, this additionally diminishes the effect of myostatin in human patients [179,180,181]. These and more contributing factors related to the lack of functional gains of myostatin inhibition has recently been reviewed [182].

Ultimately, these are all difficult hurdles to overcome. Some can be amended by improving the specificity of the modus of inhibition, others are less likely to be improved, like the expression of myostatin in fast fibers and the lack of improvement in the integrity of sarcolemma. Obviously, these factors should be taken into consideration if new targets are to be pursued.

A different issue is the goal of attempting to develop a treatment for severe diseases. In muscular dystrophies, DMD represents the pinnacle most treatments aim to improve, not only due to the frequency of patients, but also because DMD affects almost all muscles and a treatment or treatment modality for DMD may be applicable to many other muscle diseases. However, the myopic focus on finding a treatment for DMD may hurt efforts at using the very same treatments against other less severe muscle disorders. It can be argued that expecting a significant positive change in the primary outcomes may be a bit too ambitious for severe disorders. In the case of myostatin inhibition, this was tried on patients suffering from cachexia and sarcopenia, where the muscle function is unaffected by a genetic condition, without improving the muscle condition. If no myostatin inhibition treatment has been able to improve severe (DMD), intermediate (sIBM), moderate (LGMD) muscle disorders or muscle wasting related to cancer or age, then this mode of treatment is likely not suited for treating any of these disorders and conditions.

Finally, and this is an ongoing discussion throughout the entire field of treating muscle diseases, it can be argued that the primary outcome measures simply do not match the disease. It is always preferable to have a functional primary outcome measure, followed by relevant secondary outcome measures. However, the chosen functional primary outcome measure should perhaps better reflect the severity of the disease. In the absence of more flexibility of choosing the right functional outcome measure, there are usually multiple secondary outcome measures, which may demonstrate a coherent change due to treatment. However, in all clinical trials of muscular dystrophy included in this review the primary outcome measures did not demonstrate any functional improvement and the secondary outcome measures did not demonstrate any coherent improvement that could outweigh the absence of a positive primary outcome due to treatment. A recent example of a clinical trial with no improvement in functional outcome, but with a coherent improvement of secondary outcomes, is the treatment of patients with myasthenia gravis with eculizumab, which resulted in a recommendation of using eculizumab for treating this group of patients [183]. So even if the choice of functional primary outcome was less than optimal, the results of the secondary outcomes do not suggest that myostatin inhibition was a viable treatment for any of the muscle disorders and conditions in clinical trial so far in our opinion.

## 9. Future Use of Myostatin Inhibition

Over 20 years ago, the discovery of myostatin gave patients, clinicians and caretakers a hope that myostatin would provide a benchmark in treating neuromuscular disorders and that the promising results in preclinical settings would translate into clinical remission in patients. Unfortunately, the disappointing results in almost any clinical trial associated with myostatin inhibition will most likely discourage further research and development into myostatin inhibition. However, applying myostatin inhibitors as an adjuvating therapy to gene therapy restoring e.g., truncated dystrophin as previously shown in animal models [119,184] or in combination with growth factors with myotrophic properties, could introduce myostatin inhibition as a primer for the muscle fiber before salvation by antisense oligonucleotides. Milder dystrophic phenotypes with higher myostatin levels such as myotonic dystrophy [36] could possibly benefit from myostatin inhibition and muscular dystrophies characterized by proximal weakness in larger muscle groups may be a candidate for local treatment by gene therapy, as previously demonstrated [176]. After encouraging preclinical results in SMA mice, the combination treatment of myostatin inhibition and *SMN2* gene expression through a splice modulator may have more success in a clinical trial, since it aims at increasing muscle mass and correcting the functional deficit leading to SMA [133]. If the application of myostatin inhibition to the muscular dystrophies is deemed futile, the approach may be more advantageous in subjects with a healthy muscular phenotype but where other factors, such as immobilization [173], induce muscular atrophy and wasting. It may also be relevant as a treatment for insulin resistance and obesity [185,186] or for alleviating muscle wasting during future prolonged space travel in an environment with microgravity [68]. However, considering the general failure to treat human muscle diseases so far, a more specific myostatin inhibition may be required that decreases or eliminates the effect on other molecular pathways related to myostatin signaling for the continued relevance in muscle atrophy diseases.

## Figures and Tables

**Table 1:** Results of previously published data from various means of myostatin inhibition in animal models.

Species/Model	Compound	Muscle Morphology	Fiber-Type Specific Changes	Absolute Force/ Glycolytic	Specific Force/Glycolytic	Absolute Force/ Oxidative	Specific Force/Oxidative	Stress-Induced Force Drop	Histopathological Effect of Myostatin Inhibition	Reference
**Antibodies Blocking Myostatin**										
Mouse/wild-type (BALB/c, C57BL/6)	JA16, ATA-842, mRK35, YN41, muSRK-015P, GYM-mFc	Fiber CSA increased in EDL [67] and Gas Increased weight of Gas, TA, Quad and TB, plantaris, Sol [68]	Increased IIB fiber CSA, no effect on overall composition [69]	Increased grip strength [68,70,71]						[67,68,69,70,71,72]
Mouse/*mdx*, Mouse/*Sgcd^−/−^, Sgcg^−/−^*	JA16	EDL: Increased weight and single fiber area [73,74]. Increase in TA, Quad, Gas [75]		EDL: increased force	EDL: No effect			No effect	*Sgcd*^−/−^: No improvement in histopathology of TA, EDL, Gas and diaphragm albeit hydroxyproline reduced in TA. Fibrosis in diaphragm increased (*Sgcd*^−/−^ [75]) and decreased (*mdx* [73])	[73,74,75]
Mouse/*mdx*	PF-354	Increase in hindlimb muscle weight of 5 weeks treatment, no effect after 8 weeks. No effect/reduction in CSA		Diaphragm: No effect	Diaphragm increased (young)/no effect (old)			No effect	Diaphragm: Increased fiber size in young animals, decreased fiber size in old animals	[76]
Mouse/*mdx*, *TgCTA1*^D286G^, *Sod1*^G93A^, *A*17, Rat/S*od1*^G93A^ Mouse/*SmnΔ7*	mRK35/RK35 muSRK-015P (in *SmnΔ7*)	TA, Gas, Quad, EDL, diaphragm weight increased. Increased CSA in TA, EDL. No effect on weight or CSA in soleus [77]	Quad: Increased proportion of IIB fibers [78] Increase in IIB fiber CSA, no effect in remaining fiber-types [79]	TA, EDL: Increased force Plantarflexor group increased torque [79]	TA, EDL: No effect effect on plantarflexor group [79]				Gas: reduced atrophy, preserved fiber diameter. Diaphragm integrity preserved [80]. Reduced collagen I, III, IV deposits. No effect on intranuclear inclusion bodies [77,81]. Increased number of tubular aggregates [78].	[72,77,78,79,80,81]
Mouse/ CB17-SCID, C57BL/6, (Dexamethasone atrophy)	REGN1033	Increased weight in Gas and TA. Fiber area increased in Gas	No effect on fiber type composition	TA: Increased force	TA: No effect					[82]
Monkey/cynomolgus	MYO-029, Domagrozu-mab, GYM-cyfc	Increased muscular circumference								[71,72,83]
**Myostatin Propeptide Administration or Overexpression**										
Mouse/*mdx*	Recombinant propeptide-Fc	EDL: weight, CSA, single fiber area increased		EDL: Increased force	EDL: Increased force			No effect	Decreased pathological changes	[84]
Mouse/*mdx*	AAV8- MPRO76AFc	TA, Quad, Gas, Diaphragm increased		TA: Increased force	TA: No effect				Larger fibers, less fibrosis	[85]
Mouse/calpatin 3-null mice (LGMD2A), *Sgca*^−/−^ (LGMD2D)	rAAV2/1mSeAP-propmyoD76A	Increased muscle mass in calpain-3-null mice, no effect in *Sgca*^−/−^-mice		EDL: increased force (calpain-3-null mice)	EDL: No effect (calpain-3-null mice)	Soleus: Increased force (calpain-3-null mice)	Soleus: No effect (calpain-3-null mice)			[86]
**Soluble Receptor (sActRIIB-Fc)**										
Wild-type, C57BL/6 C57BL/10	ACE-031, sActRIIB, RAP-031 ACE-2494	Increased muscle weight. Fiber CSA increased in EDL [87] and in whole TA [88]	Soleus: Type I and II-fiber CSA increased [89]. Quad; increased size of I, IIA, IIB-fibers. No fiber-type switch [90]	EDL: twitch force increased, no effect on tetanic force [87]. Gas: no effect on max tetanic force [91]	EDL: no effect [92,93] Gas: decreased [91]	Soleus: increased force [92]	Soleus: no effect force [92]			[87,88,89,90,91,92,93,94,95,96]
Mouse/*mdx*	RAP-031, sActRIIB-Fc	Muscle weight increased Diaphragm and triceps myofiber increased [97]. EDL single fiber CSA increased [98].	No fiber-type conversion [92]	EDL: increased force [98,99]	EDL: increased force [98], No effect [99]. EDL, TA decreased force in older animals	Soleus decreased force	Soleus decreased force	No effect	Diaphragm, TA: No effect on histopathology, hydroxyproline [94,98]. Fibrosis decreased [97]. No visible effects on H/E pathology. SDH stains without effect of treatment [100]. eMHC: no effect [94].	[92,94,97,98,99,100]
Mouse/Tg*Acta1*^H40Y^, *Mtm1*^R69C^, *Mtm*^1δ4^, *R6/2*, *Dysf*^−/−^, *Cav3*^P104L^	RAP-031, sActRIIB-Fc	Increased muscle weight, increased fiber size	Quad: oxidative fiber diameter increased. Diaphragm: glycolytic myofibers hypertrophy [101]. IIB fiber hypertrophy, no fiber type switch [90,102]	No effect [101]. EDL, TA increased force [103]	No effect [101]	No effect [101]	No effect [101]		Nemaline rod structures unchanged [101]. Gross evaluation of diaphragm: unaffected by genotype or treatment [90]. Fibrotic changes improved	[90,101,102,103,104,105]
**Anti-ActRIIB Antibody**										
Mouse/SCID	BYM338	Increased weight of TA, EDL, Gas. Soleus increased weight (in high dose) [106]		Gas: increased force [107]						[106,107]
Mouse/C57BL/6 (glucocorticoid-induced atrophy)	BYM338	TA weight and CSA increased		TA increased force						[106]
**Follistatin Administration or Overexpression**										
Mouse/ *F66;Dysf^−/−^, F66;mdx*	Follistatin overexpression	Muscle mass maintained in *F66;mdx*, decreased in *F66;Dysf^−/−^*			*F66;Dysf^−/−^:* EDL: Decreased force				*F66;Dysf*^−/−^: Exacerbation of dystrophic features. Increased Evans Blue Dye (EBD) uptake *F66;mdx*: Dystrophic features not exacerbated, mild improvement	[104]
Mouse/*mdx*, *Sod1*^G93A^	AAV-delivered follistatin i.m.	Increased weight of TA, Gas, Quad, triceps		Increased grip strength					Young *mdx*: increased myofiber size. Satellite cell markers: no diff Old *mdx*: Fever necrotic fibers and mononuclear infiltrates	[108,109]
Monkey/Cynomolgus	AAV-delivered follistatin i.m	Increased fiber size		Quad: Increased force					Myofiber hypertrophy	[110]
Mouse/C57BL10, *mdx*,	ACE-083	Increased CSA, weight		TA: increased force	TA: no effect					[111]
Mouse/C57BL/6	FS-EEE-mFc and FST288-Fc	Increased muscle weight								[99,112]
Mouse/*mdx*	FS-EEE-mFc	Increased weight in gas, Quad, triceps, TA		EDL: Increased force	EDL: No effect				Decreased necrosis and fibrosis in Quad, no effect in diaphragm	[99]
**Liver-mediated Overexpression of Dominant-negative Myostatin (dnMSTN), sActRIIB and Myostatin Propeptide**										
Mouse/*MF*-1 (wild-type)	AAV8 over-ekspression (propeptide)	Gas, TA increased mass. EDL and soleus increased CSA.	Increased CSA of type I, IIA and IIB-fibers	EDL: No effect	EDL: No effect	Soleus: increased force	Soleus: No effect			[113]
Mouse/*Sma*^C/C^	AAV-mediated systemic expression (dnMSTN and sActRIIB)	Increased weight in TA, Gas, Quad. dnMSTN-cohort: Increased CSA in EDL and TA but not in soleus	TA: Increased IIA size EDL: Increased IIA and IIB size and total fiber number. Soleus: No effect vs. controls. I-fibers generally unaffected	EDL increased vs. SMA^C/C^ control	EDL; Decreased force	Soleus: Increased force	Soleus: No effect			[114]
Mouse/*mdx*	AAV-delivered liver-specific promoter: dnMSTN, sActRIIB	Increased weight in TA, Gas, Quad, EDL, Soleus EDL: increased CSA Soleus: No effect in weight [115]	EDL: IA + IIB increased fiber size. Increased proportion of IIB fibers in EDL and Soleus. Soleus: Increased size and proportion of IIA-fibers Diaphragm: IIX fibers proportion increased, IIA fibers proportion decrease [116] Diaphragm: No effect in specific fiber-type size [115]	EDL: increased force	No effect (decreased force by 10 months of treatment)	Soleus: increased force	Soleus increased force [116] Soleus no difference [115]. Diaphragm: no effect			[115,116,117]
Dog/GRMD	AAV-delivered liver-specific promoter (dnMSTN)	Increased weight in Tibialis cranialis, EDL, Gas, flexor digitorum superficialis	Increased size of IIA-fibers, no effect in I-fibers. No fiber type switch							[118]
**RNA Interference and Anti-oligonucleotides against Myostatin or ActRIIB**										
Mouse/*mdx*	Antimyostatin PMO	No effect in weight of diaphragm, EDL, Gas, Soleus, TA	Diaphragm: no difference in fiber-type content (I, IIA, IIX, IIB)						Diaphragm and TA: no effect on fiber diameter and collagen IV content	[119]
Mouse/*mdx* (female)	AAV-delivered shRNA, i.m.	TA: No effect on CSA, fiber number increased		TA: No effect	TA: No effect					[120]
**AAV-Cas9-mediated Myostatin Gene Editing**										
Mouse/C57/BL10	rAAV-SaCas9		Increased fiber area and number of fibers per area							[79]
**Myostatin Knock-out/Crossbreeding**										
Mouse/*Mstn^−/−^*		Increased muscle weight vs. wild-type. Increased fiber number and CSA of EDL and soleus [121]	EDL fiber-type composition: IIA and IIX incidence decreased, IIB increased in EDL and TA. Soleus CSA increased only in IIA-fibers [122]	EDL: Increased [121]/no effect [122,123]	EDL: Decreased	Soleus: Increased	Soleus: No effect	EDL: Force deficit Soleus: No force deficit	Decreased hydroxyproline content in EDL, no effect in soleus [121]. Cytoplasmic inclusions of tubular aggregates in older mice [123]	[121,122,123,124,125,126,127]
Mouse/*Beh*^C/C^		Increased muscle weight		EDL: No effect [123]	EDL: Decreased force [123]					[123,128]
Mouse/ *Mstn^−/−^;mdx* *Mstn^−/−^;Sgcd* ^−/−^ *Mstn*^Pro^*;Cav3*^P104L^		Increased mean fiber diameter and muscle weight [105,129,130]							*Mstn*^−/−^;*mdx*: Reduced fibrosis [129] *Mstn ^−/−^;Sgcd ^−/−^:* Hydroxyproline content decreased in EDL [75]	[75,105,129,130]
Mouse/*Mstn^−/−^; dy*^W^*/dy*^W^		Increased muscle mass, muscle CSA and fiber CSA. (increased mortality)	Decreased type I fiber composition						No effect on necrosis, inflammation or infiltrating cells. Less fat tissue.	[131]

Mainstream results from various antimyostatin treatments in animal models. Specific results that were distinct for a particular study and not general for all of the references have been titled as such. Abbreviations: AAV; adeno-associated virus, ActRIIB; activin receptor type IIB, CSA; cross-sectional area, EDL; m. extensor digitorum longus, eMHC; embryonic myosin heavy chain, Gas; m. gastrocnemius, GRMD; golden retriever muscular dystrophy i.m.; intra-muscular injection, LGMD; limb-girdle muscular dystrophy, Quad; m. quadriceps, SDH; succinate dehydrogenase, TA; m. tibialis anterior, TB; m. triceps brachii.

**Table 2:** Overview of published and unpublished clinical trials with myostatin inhibitors as per PubMed-U.S. National Library of Medicine and www.clinicaltrialsregister.eu and www.clinicaltrials.gov (access date 23 February 2021)

Treatment	Sponsor	Condition	Phase of Trial	Primary Outcome	Secondary Outcome	Result	Status	Reference
**Neutralizing Monoclonal Antibodies**								
MYO-029 (Stamulumab)	Wyeth	Healthy subjects	I	Safety, tolerability, PK/PD	N/A	Well tolerated	Completed	NCT# 00563810
		BMD, FSHD, LGMD (2A, 2B, 2C, 2D, 2E, 2I)	I/II	Safety	Biological activity (manual muscle test, QMT, TFT, pulmonary function test, subject-reported outcome, MRI, change in muscle mass, LBM)	Adverse effects, secondary outcome not reached	Completed	[154] EudraCT# 2004-000622-67 NCT# 00104078
PF-06252616 (Domagrozumab)	Pfizer	Healthy subjects	I	Safety and tolerability	PK/PD, DXA evaluation	Well tolerated. LBM and muscle volume increased	Completed	[155] NCT# 01616277
		DMD	I	Safety and tolerability, mean change 4-stair climb	TFT, pulmonary function tests, muscle volume, PK/PD	No significant between-group differences in any secondary clinical endpoints, terminated.	Terminated	[156] NCT# 02310763 Extension: NCT# 02907619
		LGMD 2I (FKRP)	I/II	Safety and tolerability	Muscle strength, TFTs, pulmonary function, LBM, PK, PD. Exploratory outcome: muscle fat fraction	Preliminary results on clinicaltrials.gov per January 31, 2021	Completed	NCT# 02841267
LY2495655 (Landogrozumab)	Lilly	Healthy subjects	I	“Clinically significant effect”	PK, PD, thigh muscle volume	Well tolerated	Completed	[157] NCT# 01341470
		Advanced cancer	I	Safety and tolerability	PK	Well tolerated	Completed	[157] NCT# 01524224
		Pancreatic Cancer /cachexia	II	Overall survival	Progression-free survival, tumor response, duration of response, LBM, TFT, PRO, pain	Primary outcome not reached	Completed/Terminated	[158] NCT# 01505530
		Older, weak fallers	II	Change in appendicular LBM	TFTs, gait speed, QMT, body composition, rate of falls, myostatin serum concentration	Primary outcome reached	Completed	[159] NCT# 01604408
		Osteoarthritis undergoing total hip replacement	II	Change in appendicular LBM	Secondary: QMT, PRO, whole-body- composition	Primary outcome reached	Completed	[160] NCT# 01369511
REGN1033 (Trevogrumab)/SAR391786	Regeneron/ Sanofi	Healthy subjects	I	Assessment of safety, tolerability, administration	N/A	Results not reported (both studies)	Completed	NCT# 01507402, NCT# 01720576
		Healthy subjects	I	Change in total lean mass	Safety and tolerability, appendicular lean mass	Results not reported	Completed	NCT# 01910220
		Healthy subjects	I	PK in two different formulations of drug	Safety and tolerability	Results not reported	Completed	NCT# 02741739
		Sarcopenia	II	Change in total lean body mass	AE, appendicular lean mass, gait speed, SPPB, DXA-evaluated body composition, 6MWT, QMT, TFT	Results not reported	Completed	NCT# 01963598
		sIBM	II	Change in total lean mass	AE, TFT, 6MWT, 10MWT, QMT	N/A	Withdrawn	NCT# 03710941
REGN2477 (Garetsomab, Activin A-antibody) alone and in combination with REGN1033	Regeneron	Healthy subjects	I	Safety and tolerability	Thigh muscle volume, DXA-evaluated body composition, PK	Results not reported	Completed	NCT# 02943239
SRK-015 (Apitegromab)	Scholar Rock	SMA 2, SMA 3	II	Change from Baseline in the Revised Hammersmith Scale or Hammersmith Functional Motor Scale Expanded (HFMSE)	N/A	N/A	Active per January 31 2021	NCT# 03921528
GYM329/RG 6237	Chugai Pharmaceutical/Roche	Healthy subjects (limb immobilization)	I	Thigh muscle strength	Safety and tolerability, PK, PD	Results not reported	Recruiting per January 31 2021	NCT# 04708847
**Soluble ActRIIB**								
ACE-031 (Ramatercept)	Acceleron	Healthy subjects	Ia	Safety and tolerability	PK/PD, body mass evaluation by DXA and MRI	Well tolerated	Completed	[161] NCT# 00755638
		Healthy subjects	Ib	Safety and tolerability	PK/PD	Adverse effect (epistaxis) Increased LBM and thigh muscle volume	Completed	[162] NCT# 00952887
		DMD	II	Safety and tolerability	PK/PD (MRI evaluation, bone mineral density, TFT)	Body mass, Bone mineral density MD improved vs. baseline (BL) No difference vs. placebo AE (telangiectasias, epistaxis)	Terminated	[163] NCT# 01099761 Extension: NCT# 01239758
ACE-2494		Healthy subjects	I	Safety and tolerability	PK/PD, DXA-evaluated body composition, thigh muscle volume evaluated by MRI	Development of antidrug antibodies	Terminated	[164] NCT# 03478319
**Follistatin-Fc**								
ACE-083	Acceleron	Healthy subjects	I	Safety and tolerability	PK/PD, MRI/DXA evaluation, QMT	Well tolerated	Completed	[165] NCT# 02257489
		FSH	II	Safety and tolerability	PK, PD, QMT, TFT, QOL	Did not meet functional secondary endpoint	Terminated	NCT# 02927080
		Charcot–Marie–Tooth	II	Safety, tolerability, Muscle volume estimated by MRI	PK/PD, Muscular fat infiltration, QMT, TFT, QOL, Charcot–Marie–Tooth examination score)	Did not meet functional secondary endpoint	Terminated	NCT# 03124459
**Antimyostatin Adnectin**								
BMS-986089	Bristol-Meyers-Squibb/Hoffmann-La Roche/Roche/ Greentech	Healthy subjects	I	Safety and tolerability	Pharmacokinetics	Results not reported	Completed	NCT# 02145234
RG6202/BMS-986089/ RO-7239361		DMD	Ib/II	Safety and tolerability	Thigh contractive tissue, CSA, PK	No AE. Increased LBM	Terminated	[166] NCT# 02515669
RO-7239361/RG6206		DMD	II/III	Changes in North Star Ambulatory Assessment score	TFT, QMT, 6MWT, walk, run and stride velocity	N/A	Discontinued	[167] NCT# 03039686
**Anti-ActRIIB Antibody**								
BYM-338 (Bimagrumab)	Novartis	sIBM	II	Change in muscle volume	Body composition, LBM, QMT, TFT, 6MWT	Primary outcome reached	Completed	[168] NCT# 01423110 Extension: NCT# 02250443 (terminated early)
		sIBM	IIb/III	Change in 6MWT	LBM, QMT, sIBM functional assessment, rate of falls, SPPB	Primary outcome not reached	Completed	[169,170] NCT# 01925209 EudraCT# 2013-000705-23 Extension: NCT# 02573467 EudraCT# 2015-001411-12)
		Sarcopenia	II	Change from baseline in SPPB	Safety, tolerability, 6MWT, gait speed, total LBM	Increased appendicular skeletal muscle index and LBM from baseline in 700 mg treatment cohort. No functional improvement [171]	Completed	[171] NCT# 02333331 EudraCT# 2014-003482-25 Extension: NCT# 02468674 Extension: 2015-000471-27
		Sarcopenia	II	Thigh muscle volume, intramuscular and subcutaneous fat tissue	Total LBM, QMT, TFT	Primary endpoint reached	Completed	[172] NCT# 01601600
		Patients undergoing surgical treatment of hip fracture	IIa/IIb	Change in total LBM	Gait speed, SPPB, safety and tolerability, rate of falls	Results not reported	Completed	NCT# 02152761 EudraCT# 2013-003439-31
		Casting-induced muscle atrophy (healthy)	N/A	Thigh muscle volume, change in intramuscular and subcutaneous adipose tissue	QMT, safety and tolerability	Primary endpoint reached (muscle volume)		[173] No clinical trial ID specified in article
		COPD	II	Change in thigh muscle volume	6MWT, PK	Primary endpoint reached	Completed	[174] NCT# 01669174
		Cancer cachexia (lung or pancreas)	II	Change in thigh muscle volume	Body weight, PK/PD, bone mineral density, LBM, physical activity levels	Results submitted, *p*-value not calculated	Completed	NCT# 01433263
		Type II diabetes	II	Change in body fat mass	HbA1c change, PK, body weight change, insulin resistance	Results not reported	Completed	NCT# 03005288
**Follistatin Gene Therapy**								
AAV1.CMV.FS344	Children’s Hospital/Milo Biotech	BMD	I/IIA (no placebo control)	6MWT	QMT of quadriceps, muscle histology	Primary endpoint reached (in 4 of 6 subjects)	Completed	[175] NCT# 01519349
		sIBM	I/IIa	6MWT	TFT, biopsy, Western blotting	Primary endpoint reached	Completed	[176]
rAAV1.CMV. huFollistatin344	Jerry R. Mendell/Milo Therapeutics	DMD	I/II	AE	6MWT, size of muscle fibers	Results not reported	Completed	NCT# 02354781
**Antimyostatin peptibody**								
AMG-745/PINTA 745	Amgen	Prostate cancer in patients treated with androgen deprivation therapy	I	AE, PK, DXA, QMT, SPPB, TFT	N/A	LBM increased, fat mass decreased.	Completed	[177]
		Age-associated muscle loss	II	Thigh CSA	QMT, TFT, 6MWT, PK	N/A	Withdrawn	NCT# 00975104
		End stage renal disease, kidney disease, protein energy wasting	I/II	Safety and tolerability, LBM change	LBM, appendicular lean mass, mid upper arm muscle circumference, TFT, 6MWT	Results not reported	Completed	NCT# 01958970
**Myostatin Inhibition (Information on Myostatin Inhibition Strategy not Available)**								
BLS-M22	BioLeaders Corporation	Healthy subjects	I	Safety and tolerability	PK, immunogenicity, changes in muscle mass	Results not reported	Recruiting	NCT #03789734

Abbreviations: 10MWT; 10-min walking test, 6MWT; 6-min walking test, AE; Adverse events, BMD; Becker Muscular Dystrophy, COPD; chronic obstructive pulmonary disorder, CSA; Cross-sectional area, DMD; Duchenne Muscular Dystrophy, DXA; Dual-energy X-ray absorption, FSHD; Facio-scapulo-humoral dystrophy, LBM; Lean body mass, MRI; Magnetic resonance imaging, N/A; Not available, PRO; Patient reported outcome, PD; Pharmacodynamics, PK; Pharmacokinetics, QMT; Quantitative muscle testing, QOL; Quality of life, sIBM; spontaneous inclusion body myositis, SMA; spinal muscle atrophy, SPPB; Short Physical Performance Battery, TFT; Timed function test.

## Supplementary Materials

The following are available online at https://www.mdpi.com/2073-4409/10/3/533/s1: Supplementary Table S1: Individual results of published data from studies of myostatin inhibition in animal models; Supplementary Table S2: Detailed overview of published and unpublished clinical trials with myostatin inhibitors as per PubMed-U.S. National Library of Medicine, www.clinicaltrialsregister.eu and www.clinicaltrials.gov (access date 23 February 2021).
