# Supplementary material for: Antimyostatin Treatment in Health and Disease: The Story of Great Expectations and Limited Success
Source: Cells. 2021 Mar 3;10(3):533. doi: 10.3390/cells10030533 (PMC8001237; doi:10.3390/cells10030533)
Supplement: Supplementary file 1 [file cells-10-00533-s001.pdf]

Supplementary table 1: Individual results of published data from studies of myostatin inhibition in animal models.

| Species/model (condition) | Compound, (delivery) | Age at treatment start | Length of treatment or age at exam if knock-out | Body weight | Muscle morphology | Fiber-type specific changes | Absolute force/ glycolytic | Specific force/ glycolytic | Absolute force/ oxidative | Specific force/ oxidative | Stress-induced force drop | Creatine kinase | Histopathological effect of myostatin inhibition | Reference |
|---|---|---|---|---|---|---|---|---|---|---|---|---|---|---|
| **Antibodies blocking myostatin** | | | | | | | | | | | | | | |
| Mouse/ C57BL/6 (male) | JA16 | 5-8 weeks | 15 weeks | 110 % vs control, still growth phase | Increased weight in Quad and Gas | | | | | | | | | [1] |
| Mouse/ BALB/c (female) | | 7-8 weeks | 4 weeks | | Increased fiber size in EDL | | | | | | | | | |
| Mouse/ BALB/c (female) | | | 8 weeks | | Increased weight in Gas and Quad | | | | | | | | | |
| Mouse/ wild-type | ATA 842 | 10 weeks | 4 weeks | Increased | Increased weight in Gas | | Grip strength increased | | | | | | | [2] |
| Mouse/ *mdx* | JA16 | 4 weeks | 3 months | Increased | Increased weight and single fiber size in EDL | | EDL twitch + tetanic force increased | EDL: No effect | | | No effect of treatment | Decreased | Diaphragm: Less fibrosis and cellular infiltration | [3] |
| Mouse/ *Sgcg*$^{-/-}$ (LGMD 2C) | JA16 | 4 weeks | 3 months | No difference | Increased weight and single fiber size in EDL | | EDL twitch + tetanic force increased | EDL: No effect | | | No effect of treatment | No effect | Hydroxyproline reduced in TA but not diaphragm and EDL. Histopathology in all three muscles without improvement. | [4] |
| Mouse/ *Sgcd*$^{-/-}$ (LGMD 2F) | JA16 | 4 weeks | 3 months | | Increased weight in Gas, Plantaris, TA and Quad | | | | | | | | Gas and diaphragm: No difference in fibrosis. | [5] |
| | | 20 weeks | 3 months | | Increased weight in TA and Quad | | | | | | | | Gas and diaphragm: Fibrosis increased | |
| Mouse/ *mdx* | PF-354 | 16-17 days | 5 weeks | No difference | Increased weight in EDL Soleus, TA, Gas and Quad. Diaphragm increased size. | | Diaphragm: no effect | Diaphragm: increased force | | | No effect of treatment | | Reduced connective tissue infiltration | [6] |
| | | 12 weeks | 5 weeks | Increased | Increased weight in EDL, plantaris, TA, Gas and Quad No size improvement | | | | | | | | | |
| | | 12 weeks | 8 weeks | No difference | No difference vs controls. Reduced fiber size in Diaphragm | | Diaphragm: no effect | Diaphragm: no effect | | | No effect of treatment | | Diaphragm; no positive effect of treatment. | |
| Mouse/ SCID/ female | MYO-029 | 8 weeks | 12 weeks | | Increased weight in EDL | EDL: No difference in metabolic profile or MCH expression | | | | | | | | [7] |
| Mouse/ SCID/ female | MYO-029 | N/A | 4 weeks | | Fiber hypertrophy in EDL (data not shown) | | | | | | | | | [8] |
| Monkey/ Cynomolgus | | N/A | 39 weeks | | Increased muscular circumference | | | | | | | | | |
| Mouse/ Tg*Acta1*$^{D286G}$ (nemaline myopathy) | mRK35 | 2 weeks | 6 months | Increased | Increased weight of Triceps, Quad and Gas, no difference in EDL. Quad fiber size increased | Quad: Increased proportion of IIB fibers | EDL: increased force | EDL: no effect | | | | | Increased number of tubular aggregates | [9] |
| Mouse/ wild-type | mRK35 | 8 weeks | 4 weeks | Increased | Increased weight in Quad and Gas | | | | | | | | | [10] |
| | | 1 year | 4 weeks | No difference | Increased weight in TA and Quad. No difference in Gas | | | | | | | | | |

| | | | | | | | | | | | | | | |
|---|---|---|---|---|---|---|---|---|---|---|---|---|---|---|
| Mouse/ *mdx* | | 8 weeks | 8 weeks | Increased | Increased weight in TA, Gas, Quad, EDL. Increased CSA in EDL | | EDL: increased force | EDL: no effect | | | | | | |
| | | | 4 weeks | | Increased fiber size in TA | | | | | | | | | |
| Monkey/ cynomolgus | Domagrozumab (PF-06252616) | 3-5 years | 8 weeks | | Axial and appendicular muscle mass increased (per MRI) | | | | | | | | | |
| Mouse/ $Sod1^{G93A}$ (ALS) | RK35 | 4 weeks | 84-90 days (end-stage˜135) | | Increased weight in Gas, Diaphragm, Quad, TA | | Increased grip strength | | | | | | Gas: reduced atrophy, preserved fiber diameter. Diaphragm integrity preserved | [11] |
| Rat/ $Sod1^{G93A}$ (ALS) | | 6 weeks | 95 days (end-stage ˜ 128) | | Increased weight in Gas, Diaphragm, Quad and TA | | Increased grip strength | | | | | | | |
| Mouse/ *A17* (OPMD) | RK35 | 12 weeks | 10 weeks | Increased | Increased weight and CSA in TA and EDL. No effect in soleus | | TA; Increased force | TA: No difference | | | | | TA: Reduced collagen I, III, IV deposits. No effect on intranuclear inclusion bodies. | [12] |
| | | 42 weeks | 10 weeks | No difference | No difference in CSA or muscle weight of TA | | | | | | | | TA; No effect on intranuclear inclusion bodies. Reduced collagen IV depostion | [13] |
| Mouse/ CB17-SCID | REGN1033 | 9 weeks | 4 weeks | Increased | Increased weight in Gas and TA. Increased fiber size of Gas | No difference in fiber-type composition | | | | | | | | [14] |
| Mouse/ C57BL/6 | | N/A | 4 weeks | | Increased weight in Gas and TA | | | | | | | | | |
| | | N/A | 3 weeks | | | | TA: increased force | TA: no effect | | | | | | |
| | | 24 months | 3 weeks | | | | TA: increased force | TA: no effect | | | | | | |
| Mouse/C57BL/6/ (Dexamethason. atrophy) | | 11 weeks | 2 weeks | | | | TA: increased force | TA: no effect | | | | | | |
| Mouse/ BALB/cAnNTac (wild type) | YN41 (ground) | 12 weeks | 6 weeks | Increased | Increased weight in TB, TA, EDL, Gas, Quad, plantaris. “Lower limb hindlimb muscle” increased CSA | | Increased grip strength | | | | | | | [15] |
| | YN41 (microgravity) | | | Increased | Increased weight in TB, Gas, Quad, TA, Plantaris, Sol. “Lower limb hindlimb muscle” increased CSA | | Increased grip strength | | | | | | | |
| Mouse/ C57BL6 | muSRK-015P | 10 weeks | 4 weeks | | Increased weight of Gas, EDL.Total CSA increased in plantarflexor group | Increased IIB fiber size, no effect on overall composition | EDL; no effect | | | | | | | [16] |
| Mouse/ *Smn∆7* (SMA) | muSRK-015P (with SMN-C1 splice modulator) | 24 days (‘late’ restoration cohort) | 4 weeks | No difference | Increased muscle mass in TA, Gas. ‘Plantarflexor muscle group’ increased CSA | Trending increase in fiber size of IIB. No difference in type I, | Plantar-flexor group: Increased | No effect on plantar-flexor | | | | | | [17] |

| | | | | | | | | | | | | | | |
|---|---|---|---|---|---|---|---|---|---|---|---|---|---|---|
| | | | | | | IIA, IIX size or overall compisition. | muscle torque | group torque | | | | | | |
| | | 24 days ('early' restoration cohort) | 4 weeks | Increased | As 'late' group | Significant increase in fiber size of IIB. No effect on type I, IIA, IIX or overall composition | As 'late' group | As 'late' group | | | | | | |
| Mouse/C57BL/6 | GYM-mFc, i.v, i.p.(murinized GYM329) | 79 weeks | 4 weeks | | | | Increased grip strength | | | | | | | [18] |
| Mouse/*mdx* | | 5 weeks | 4 weeks | Increased | Increased muscle mass | | Increased grip strength | | | | | | | |
| Mouse/SCID and 2 weeks of hindlimb suspension | | 9 weeks | 2 weeks | | | | Increased grip strength | | | | | | | |
| | GYM329 i.v. | 9 weeks | 2 weeks | | | | Increased grip strength | | | | | | | |
| Monkey/ Cynomolgus | GYM-cyFc (cynomolgus equivalent).v. | 3 years | 2 months | Increased | Increased muscle section area per MRI | | | | | | | | | |
| **Myostatin propeptide administration or overexpression** | | | | | | | | | | | | | | |
| Mouse/ SCID | Myostatin Propeptide (D76A) | Young | 5 weeks | Increased | Increased weight in Gas | | | | | | | | | [19] |
| Mouse/ *mdx* | Recombinant propeptide-Fc | 4 weeks | 3 months | Increased | Increased weight and single fiber area in EDL | | EDL: increased force | EDL: increased force | | | No effect of treatment | Decreased | Diaphragm: Decreased pathological changes | [20] |
| Mouse/ *mdx* | AAV8-MPRO76AFc | 3 months | 4 months | Increased | Increased weight of TA, Gas, Quad, Diaphragm | Fiber radius increased | TA: Absolute twitch and tetanus increased | TA: No effect | | | | Decreased | Larger fibers, less fibrosis and fewer mononuclear cells | [21] |
| Mouse/ C57/B10 | AAV8-MPRO | 3-days old | | Increased | | | TA: No effect | TA: No effect | | | | | | |
| | AAV8-MPRO76AFc | 4 months old | | No effect | | | | | | | | | | |
| Mouse/ 129SV (wild-type) | rAAV2/1mSeAP-propmyoD76A, i.m. | 2 months old | 6 weeks | | Increased muscle mass and fiber size of injected muscle | | | | | | | | | [22] |
| Mouse/ calpain-3 deficient mice (LGMD2A) | | 5 months | ? (intra-arterial injections) | No effect | Increased muscle mass of EDL, Gas, Quad | | EDL: Increased force | EDL: No effect | Soleus: Increased force | Soleus: No effect | | | | |
| | | 2 months | 6 weeks | | Increased muscle mass, increased single fiber area but not number | | Improved | | | | | | No improvement of histology | |
| Mouse/ *Sgca*$^{-/-}$ (LGMD2D) | | 5 weeks – 2 months | 6 weeks | | No increase in muscle mass | | | | | | | | No effect on stains for alkaline phosphatase as measurement for successful gene transfer | |
| **Soluble receptor (sActRIIB-Fc)** | | | | | | | | | | | | | | |
| Mouse/*mdx* | RAP-031 (1 mg/kg) | 4 weeks | 12 weeks | No difference | Increased weight of Gas. No difference in weight of TA and Quad. Increased weight but not CSA in EDL | | EDL: increased force | EDL: increased force | | | No effect | Decreased | Diaphragm, TA: No difference in histopathology, hydroxyproline | [23] |
| | RAP-031 (10 mg/kg) | | | Increased | Increased weight in Gas, TA, Quad, EDL. Increased single fiber size in EDL. | | EDL: increased force | EDL: no effect | | | No effect | Decreased | Diaphragm, TA: No difference in histopathology, hydroxyproline | |
| Mouse/ *mdx* | RAP-031 | 4 weeks | 30, 60, 90 days | | | | | | | | | | | [24] |
| Mouse/ C57BL/6 | RAP-031 | 8 weeks | 4 weeks 10 mg/kg 2x/wk | Increased | Increased weight in Gas, EDL, Quad. | | EDL: increased | | | | | | | [25] |

| | | | | | | | | | | | | | | |
|---|---|---|---|---|---|---|---|---|---|---|---|---|---|---|
| | | | | | Fiber size increased in EDL. | | force (twitch force)<br>EDL: No effect (tetanic force) | | | | | | | |
| | | | 10 weeks | No effect | | | | | | | | | | |
| Mouse/ C57BL/6 | RAP-031 | 8 weeks | 5 weeks | Increased | | | Gas: increased force (twitch force)<br>Gas: No effect (tetanic force) | Gas: no effect | | | | | | [26] |
| Mouse/ C57BL/6 | RAP-031 | 3 months | 8 weeks | Increased | Increased volume of Gas. | | Gas: no effect | Gas: decreased force | | | | | | [27] |
| Mouse/ *Acta1*$^{H40Y}$ | RAP-031 | 14 days | 3½ months | Increased | Increased weight of Triceps, Gas, Quad.<br>Overall larger myofiber size | Quad: oxidative fiber diameter increased.<br>Diaphragm: glycolytic myofibers hypertrophy | EDL: no effect | EDL: no effect | Soleus: no effect | Soleus: no effect | | | No decrease in nemaline rod structures | [28] |
| Mouse/ *Neb* cKO | RAP-031 | 14 days | 7 weeks | No effect | No effect. No difference in CSA, fiber size or weight | Not quantified | | | | | | | No effect | [29] |
| Mouse/ *Mtm1δ4* | RAP-031 | 2 weeks | Until death (mean 54 days for treated animals) | Increased | Increased weight in Gas, Quad, triceps | Quad; Hypertrophy only in IIB fibers.<br>No fiber-type switch | | | | | | | Gross evaluation of diaphragm: unaffected by genotype or treatment | [30] |
| Mouse/ C57BL/6 | | | Corresponding to Mtm1δ4 life-span | Increased | Increased weight in Quad, triceps, no difference in Gas.<br>Increased fiber size of Quad | Quad; Increased size: I, IIA, IIB.<br>No fiber-type switch | | | | | | | | |
| Mouse/ *Mtm1*$^{R69C}$ | RAP-031 | 2 weeks | 5½ months | | Increased weight Gas.<br>No difference in Quad and triceps.<br>Increased average fiber diameter in Gas | IIB fibers increased size.<br>No fiber-type switch | | | | | | | | [31] |
| Mouse/ *mdx* | RAP-031 | 8 weeks | 16 weeks | Increased | Increased weight in Quad, Gas, TA, Plantaris, EDL. No difference in Soleus.<br>Increased small fiber profiles in EDL. | No fiber-type conversion in either EDL or soleus | EDL: no effect | EDL: decreased force | Soleus: decreased force | Soleus: decreased force | | No effect | | [32] |
| Mouse/ C57BL/6 | | | | Increased | Increased weight in Quad, Gas, TA, Plantaris and EDL.<br>No difference in soleus | | EDL: increased force | EDL: no effect | Soleus: increased force | Soleus: no effect | | | | |
| Mouse/ *mdx* | RAP-031 | 3 months | 8 weeks | Increased | Increased volume of Gas | No change in MHC isoforms | Gas: increased force | Gas: no effect | | | | | | [33] |
| Mouse/ C57BL/6 | ACE-031 | 8 weeks | 4 weeks | Increased | Increased weight in soleus, plantaris, Gas, EDL | Soleus: Type I and II-fiber size increased | | | | | | | | [34] |
| Mouse/ C57BL/6 | sActRIIB | 3 months | 8 weeks | Increased | Increased volume in Gas. | | | | | | | | | [35] |
| Mouse / C57BL/6 | sActRIIB-Fc, i.m. | | | | Increased weight in Gas, TA, pectoralis, Quad | | | | | | | | | [36] |
| Mouse/ C57BL/10 | sActRIIB-Fc | 6 weeks | 2 weeks | Increased | Increased weight in Gas, Quad, TA | | | | | | | | | [37] |
| Mouse/ *mdx* | | 8 weeks | 5 weeks | Increased | Increased weight in EDL, Gas, TA,<br>fiber size increased in TA | | TA: no effect | TA: decreased force | | | No effect | | No effect in histopathology.<br>eMHC: no effect | |

| | | | | | | | | | | | | | | |
|---|---|---|---|---|---|---|---|---|---|---|---|---|---|---|
| Mouse/ *mdx* | sActRIIB-Fc | 2 years | 6 weeks | No difference | Increased weight in Quad, Tri, diaphragm.<br>Increased myofiber size in Diaphragm and Triceps | | | | | | | | Fibrosis, lipid infiltration and hydroxyproline content decreased in limb and diaphragm<br>Triceps: eMHC increased | [38] |
| Mouse/ *R6/2* | sActRIIB-Fc | 5 weeks | 6 weeks | No difference | Increased weight in Quad, Gas, TA.<br>Increased fiber size of TA and Quad | | EDL, TA: increased force | | | | | | | [39] |
| Mouse/ C57BL/10 Hypoxia atrophy model | sActRIIB-Fc | 10 weeks | 2 weeks followed by 2 weeks of hypoxia | Increased | Increased weight in Soleus, EDL, Gas, TA and Quad.<br>Increased whole-muscle and single fiber size in EDL | | EDL, increased force | | | | Improved resistance to eccentric lengthening | | | [40] |
| Mouse/ C57BL/6 (orchidecctomized) | sActRIIB-Fc | 5-6 months | 30 days | | Increased weight in Gas, TA and Quad.<br>size increased in TA | | | | | | | | | [41] |
| Mouse/*mdx* | sActRIIB-Fc | 6-7 weeks | 7 weeks | Increased | Increased weight in Gas, soleus, Quad, EDL and TA | | | | | | | No effect | No visible differences in pathology on H/E. SDH stains without effect of treatment | [42] |
| Mouse/ $Dysf^{-/-}$ | ActRIIB-Fc | 6 weeks and 7 months | 4 weeks | | | | | | | | | Increased | Decreased fibrosis | [43] |
| Mouse/ *mdx* | sActRIIB-mFc | 3 weeks | 12 weeks | Increased | Increased weight in Gas, Quad, triceps and TA | | EDL: increased force | EDL: no effect | | | | No effect | | [44] |
| Mouse/ $Cav3^{P104L}$ | sActRIIB-Fc | 6 weeks | 2 weeks | | Increased myofiber area in triceps and Quad | | | | | | | | | [45] |
| Mouse/ wild type | ACE-2494, s.c. | 8 weeks | 4 weeks | | Increased weight in TA, Quad, Gas and EDL. No effect of weight in soleus | Sol fiber size increased | | | | | | | | [46] |
| Mouse/ *SmnΔ7* | sActRIIB-Fc | Postnatally | 15 days | | Increased muscle weight of Gas, Quad and triceps | | | | | | | | | [47] |
| **Anti-ActRIIB-antibody** | | | | | | | | | | | | | | |
| Mouse/ SCID | BYM338 | 12 weeks | 4 weeks | Increased | | | Gas: increased force | | | | | | | [48] |
| Mouse/ SCID | BYM338 | 10 weeks | 4 weeks 6 mg/kg | Increased | Increased weight in TA, EDL and Gas, no difference in soleus. | | | | | | | | | [19] |
| | | | 4 weeks 20 mg/kg | Increased | Increased weight in TA, EDL, Gas and soleus | | | | | | | | | |
| Mouse/ C57BL/6 (glucocorticoid-induced atrophy) | | 4 months old | 2 weeks concurrent with dexamethasone | Increased | Increased weight and CSA of TA | | TA: increased force | | | | | | | |
| | | | 2 weeks post 3 weeks dexamethasone treatment | | Increased weight in Gas, plantaris and TA | | | | | | | | | |
| Mouse/SCID | | Young | 5 weeks | Increased | Increased weight in Gas | | | | | | | | | |
| Mouse/ $Mstn^{Ln/Ln}$ | CDD866 | 19-24 weeks | 4 weeks | Increased | Increased muscle weight (not specified) | | | | | | | | | |
| **Follistatin administration or overexpression** | | | | | | | | | | | | | | |
| Mouse/ *F66;Dysf*$^{-/-}$ (LGMD2B) | Follistatin over-expression crossed with $Dysf^{-/-}$ mice | | 8 months | | Muscle mass decreased with age | | | EDL: decreased force | | | Eccentric injury performed but not described | Increased, then decline with age | Exacerbation of dystrophic features.<br>Increased Evans Blue Dye (EBD) uptake | [43] |
| Mouse/ F66;*mdx* | | | | | Muscle mass maintained | | | | | | | | Dystrophic features not exacerbated, mild improvement | |

| | | | | | | | | | | | | | | |
|---|---|---|---|---|---|---|---|---|---|---|---|---|---|---|
| Mouse/ *SMAΔ7* | Follistatin over-ekspression | | | | Increased weight of Quad, none in Gas or triceps | | | | | | | | | [47] |
| Mouse/ C57BL/6 | AAV-delivered follistatin i.m. | 4 weeks | Evaluation at 180 days | Increased | Increased weight in TA, Gas, Quad, Triceps | | | | | | | | | [49] |
| Mouse/ *mdx* | | 3 weeks | 5 months | Increased | Increased weight in TA, Gas, Quad, Triceps | | Increased grip strength | | | | | Decreased | Myofiber size increased. Satellite cell markers: no diff | |
| | | 210 days | 560 days | | | | Increased grip strength | | | | | | Fewer necrotic fibers and mononuclear infiltrates | |
| Monkey/ Cynomolgus | AAV-delivered follistatin i.m | Not specified | Single administration of three injections in right Quad | | Increased fiber size in Quad | | Quad: Increased force | | | | | | Myofiber hypertrophy | [50] |
| Mouse/ C57Bl10 | ACE-083, i.m. | Not specified | 4 weeks | | Physiological CSA and weight of TA increased, fiber hypertrophy | | TA: increased | TA: no effect | | | | | | [51] |
| Mouse/ Trembler-J (Charcot-Marie-Tooth) | | 7 months | 4 weeks | No effect | Physiological CSA and weight of TA increased, fiber hypertrophy | | TA: increased | TA: no effect | | | | | | |
| Mouse/ *mdx* | | 4 weeks | 4 weeks | No effect | Physiological CSA and weight of TA increased, fiber hypertrophy | | TA: increased | TA: no effect | | | | Reduced (not statistically significant) | | |
| Mouse/ C57BL/6 | Follistatin fusion-protein (FST288-Fc) i.m. or s.c. | 9 weeks | 4 weeks | | Increased weight in Gas vs uninjected muscle | | | | | | | | | [36] |
| Mouse/ C57BL/6 | Engineered Follistatin (FS-EEE-mFc) i.v. or s.c. | 9-10 weeks | 4 weeks | Increased | Increased weight in Gas, Quad, triceps and TA | | | | | | | | | [44] |
| Mouse/ *mdx* | | 3 weeks | 12 weeks | Increased | Increased weight in Gas, Quad, Triceps and TA | | EDL: increased force | EDL: no effect | | | | No effect | Decreased necrosis and fibrosis in Quad, no difference in diaphragm | |
| Mouse/ *hSMN2*$^{+/+}$; *hSMNΔ7*$^{+/+}$; *Smn*$^{-/-}$ | Recombinant human follistatin | | Untill postnatal day 16 | | Some effect in TA and Gas but none at treatment day 16. No effect on CSA | | | | | | | | | [52] |
| Mouse/ C57BL/6; *FS I-I* | Follistatin overexpression (transgenic mice) | | 5, 9, 13 weeks | | Increased mass, fiber no. and fiber area of EDL, Sol, Quad, TA. | | Increased grip strengh | | | | | | | [53] |
| Mouse/ *mdx;FS I-I* | | | 5, 9 weeks | | Larger muscles | | Increased grip strength | | | | | | Reduced macrophage infiltration and fibrous changes of diaphragm | |
| Mouse/ *Sod1*$^{G93A}$ (ALS) | AAV9-delivered follistatin i.m. | 40 days | Approximately 80 days | | Increased muscle mass in injected and non-injected muscles. Increased myofiber number and diameter | | Grip strength increased | | | | | | | [54] |
| **Liver-mediated overexpression of dominant-negative myostatin (dnMSTN), sActRIIB, myostatin propeptide and GASP1** | | | | | | | | | | | | | | |
| Mouse/ MF-1 (wild type) | AAV8-mediated overekspression (propeptide) | Injected at 6 weeks of age | 4- and 10-weeks post injections | Increased | Increased weight of TA and Gas, increased CSA of EDL and Sol | Increased size of type I, IIA and IIB fibers | EDL: no effect | EDL: no effect | Sol: Increased force | Sol: No effect | | | | [55] |
| Mouse/ *Sma*$^{C/C}$ | AAV-mediated systemic expression (sActRIIB) | 4 weeks | 8 weeks | Increased | Increased weight in Quad, TA, EDL, Gas. No difference in weight of soleus | TA: Increased IIA size. EDL: Increased IIA and IIB size, increased total fiber number. Soleus: No difference | EDL, TA increased force | EDL, TA: decreased force | Soleus; increased force | Soleus: no effect | | | | [56] |
| | AAV-mediated systemic expression (dnMSTN) | | | Increased | Increased weight in Quad, TA, EDL, Gas and soleus. Increased fiber size in EDL and TA but not in soleus | TA: Increased size of IIA and IIB fibers. | EDL: increased force | EDL: decreased force | Soleus: no effect | Soleus: no effect | | | | |

| | | | | | | | | | | | | | | |
|---|---|---|---|---|---|---|---|---|---|---|---|---|---|---|
| | | | | | | EDL: Increased IIA and IIB size, increased total fiber no.<br>Soleus: No effect vs controls.<br>I-fibers unaffected | | | | | | | | |
| Mouse/ *mdx* (B10 background) | AAV-mediated systemic expression (sActRIIB) | 6 weeks | 5 months | Increased | Increased weight in Quad, TA, EDL and Gas<br>Increased CSA in EDL.<br>No difference in weight or CSA in soleus | EDL: increased fiber size.<br>Soleus: no difference in fiber size.<br>Diaphragm: no difference in specific fiber-type size | EDL: increased force | EDL: no effect | Soleus: no effect | Soleus and diaphragm: no effect | | Decreased | | [57] |
| Mouse/ *D2.mdx* | AAV-delivered liver-specific promoter (dnMSTN) | 6 weeks | 16 weeks | No difference | Increased absolute mass of Gas and Quad and CSA of EDL | | EDL: increased force | EDL: no effect | | | | | | [58] |
| Mouse/ C57BL6 | AAV-delivered liver-specific promoter (dnMSTN) | Neonate | 3 months | No difference | Increased weight in Quad, TA, EDL.<br>EDL: increased fiber size<br>No difference in weight of soleus and hearts | Soleus: IIB proportion increased | EDL: increased force | EDL: no effect | | | | | | [59] |
| Mouse/ *mdx* | | Neonate | 11 months | Increased | Increased weight in TA, Gas, Quad, EDL and soleus.<br>Increased CSA in EDL and Soleus | EDL: Proportion of IIB fibers increased.<br>Soleus: IIA-proportion increased | EDL: no effect | EDL: no effect | Soleus: increased force | Soleus: no effect | | No effect | | |
| | | 1 month | 3 months | Increased | Increased weight in TA, Gas, Quad, EDL, soleus.<br>Increased CSA in EDL. | EDL: IIA + IIB increased fiber size, proportion of IIB increased.<br>Soleus: IIA increased fiber size, proportion of IIB fibers increased.<br>Diaphragm: IIX fibers proportion increased, IIA fibers proportion decrease | EDL: increased force | EDL: no effect | Soleus: increased force | Soleus: increased force<br>Diaphragm : no effect | | Decreased | | |
| | | 1 month | 10 months | Increased | Increased weight in TA, Gas, Quad, EDL and soleus<br>Increased CSA of EDL and Soleus. | EDL: Proportion of IIB fibers increased<br>Soleus: Increased IIA fiber size and proportion | EDL: increased force | EDL: decreased force | Soleus: increased force | Soleus: increased force<br>Dia-phragm: no effect | | No effect | Diaphragm: no difference in fibrosis | |
| Mouse/ *Smn2* (SMA) | AAV-MPRO (myostatin propeptide) | Birth | - | Increased | | | | | | | | | | [60] |
| | AAV-MPRO in combination with PMO versus *SMN2*-gene | | | | Increased weight of TA and Gas and muscle diameter | | | | | | | | No effect of myostatin inhibtion on CNF in TA.<br>NMJ end-plate increased area | |
| Dog/ GRMD | AAV-delivered liver-specific promoter (dnMSTN) | 10 months | 13 months | Increased | Increased mass of Tibialis cranialis, EDL, Gas, flexor digitorum superficialis | Tibialis cranialis: IIA-fibers increased size, I-fibers: no difference in size.<br>No fiber type switch in EDL or tibialis cranialis | | | | | | Decreased | | [61] |
| Mouse | AAV-mediated systemic expression | 12 weeks | | Increased | Increased myofiber size.<br>Increased mass of Gas, rectus femoris and pectoralis major. | Increased fiber diameter in fiber type I, IIA, IIB | | | | | | | | [62] |

| | | | | | | | | | | | | | | |
|---|---|---|---|---|---|---|---|---|---|---|---|---|---|---|
| **RNA interference and anti-oligonucleotides against myostatin or ActRIIB** | | | | | | | | | | | | | | |
| Mouse/ C57BL10 | Antimyostatin AOs | 6 weeks | 1,2,4,8 weeks | | TA: Increased weight after 4 weeks, increased CSA only in Vivo-PMO-mstn-treatment) | | | | | | | | No major histological changes | [63] |
| Mouse/ *mdx* | Antimyostatin AOs | 6 weeks | 8 weeks | | Increased TA weight and proximal CSA | | | | | | | | No toxicity of treatment | |
| Mouse/ *mdx* | Antimyostatin AOs | 6 weeks | 10 weeks | No difference | No difference in weight of diaphragm, EDL, Gas, Soleus, TA | Diaphragm: no difference in fiber-type content (I, IIA, IIX, IIB) | | | | | | | Diaphragm and TA: no difference in fiber diameter and collagen IV content | [64] |
| Mouse/ C57BL10 | Antimyostatin AOs | Not stated | 5 weeks | | Soleus increased mass and CSA, EDL did not | | | | | | | | | [65] |
| Mouse/ *mdx* (female) | AAV-delivered shRNA, i.m. | 6-8 weeks | 1 month | | Fiber number increased in TA, no difference in CSA | | TA: no effect | TA: no effect | | | | | | [66] |
| **AAV-Cas9-mediated myostatin gene editing** | | | | | | | | | | | | | | |
| Mouse/ C57/BL10 | rAAV–SaCas9 | 18 months | 8 weeks | | Increased fiber area and number of fibers per area | | | | | | | | | [67] |
| **Myostatin knock-out/crossbreeding** | | | | | | | | | | | | | | |
| Mouse/ *Mstn*$^{-/-}$ | | 2, 3, 4, 5 months | | Increased | All examined muscles increased weight | | | | | | | | | [68] |
| Mouse/ *Mstn*$^{-/-}$ | | | 10-12 months | | Increased wet weight, CSA and fiber number in EDL and soleus | | EDL: increased | EDL: decreased force | Soleus: increased force | Soleus: no effect | EDL: greater force deficit vs wild-type<br>Soleus: no effect | | EDL: Hydroxyproline content decreased. Soleus: no difference | [69] |
| Mouse/ *Mstn*$^{-/-}$ | | | 2 months | | Increased weight in EDL | EDL Fiber-type incidence:<br>IIA decreased<br>IIX decreased<br>IIB fibers increased | EDL: no effect | EDL: decreased force | | | | | No cytoplasmic inclusions | [70] |
| | | | 7 months | | Increased weight and CSA in EDL | | EDL: no effect | EDL: decreased force | | | | | Cytoplasmic inclusions of tubular aggregates | |
| | | | 10 months (female) | | Increased weight in EDL | | EDL: no effect | EDL: decreased force | | | | | | |
| Mouse/ *Mstn*$^{-/-}$ | | | 9 weeks | Increased | Increased weight in TA and Plantaris | IIB fiber size increased (EDL + plantaris)<br>IIA proportion decreased (plantaris only) | | | | | | | | [71] |
| Mouse/ *Mstn*$^{-/-}$ | | | 6 months | | | Percentage of type I and IIA decreased in biceps femoris and TA.<br>IIX/B increased in both muscles | | | | | | | | [72] |
| Mouse/ *Mstn*$^{-/-}$ | | | 12 weeks | Increased | Increased weight in Gas, biceps femoris, TA, EDL, soleus and Plantaris (corrected to tibia length) | Tibialis anterior: Proportion of IIB-fibers increased | | | | | | | | [73] |
| Mouse/ *Mstn*$^{-/-}$ | | | 6 months | | Increased weight in EDL and Soleus | CSA increased in EDL and soleus (in soleus; only IIA-fibers) | EDL: no effect | EDL: decreased force | Soleus: increased force | Soleus: no effect | | | | [74] |
| Mouse/ *Mstn*$^{-/-}$ | | | 6 months | | | Soleus: decreased proportion of type I, increased type II | | | | | | | | [7] |

| | | | | | | | | | | | | | | |
|---|---|---|---|---|---|---|---|---|---|---|---|---|---|---|
| | | | | | | fibers. EDL: increased proportion of glycolytic fibers RT-PCR:<br>Soleus: MHC-IB percentage decreased, IIX+IIB increased.<br>EDL: Increased proportion of IIB, decreased IIX | | | | | | | | |
| Mouse/ *Beh*$^{C/C}$ (Compact) | | | 2 months | | Increased weight in EDL | | EDL: no effect | EDL: decreased force | | | | | | [70] |
| Mouse/ *Beh*$^{C/C}$ (Compact) | | | 3-4 months, | | Increased weight in TA, Gas, Quad | | | | | | | | | [75] |
| Mouse/ *Cmpt* (Compact) | | | | | TA: Fibertype switch to IIB<br>EDL; IIB hypertrophy.<br>Sol: I and IIA hypertrophy and fiber-type switch | | | | | | | | | [76] |
| Mouse/ *Mstn* $^{-/-}$*;mdx* | | | 3, 6, 9 months (exam.) | | Mean fiber diameter increased In TA | | Improved forearm grip-force | | | | | No effect | Diaphragm: Reduced fibrosis | [77] |
| Mouse/ *Mstn* $^{-/-}$*;mdx* | | | 18 months (exam.) | | Increased weight in triceps, pectoralis, Quad, Gas. | | Improved forearm grip-force | | | | | | Diaphragm: ”Abundant myofibers” | [78] |
| Mouse/ *Mstn*$^{-/-}$*; dy*$^{W}$*/dy*$^{W}$ | | | 3 weeks | No difference | Larger muscle mass and fiber numbers in rectus femoris | Reduced type I fibers in rectus femoris | | | | | | | No effect on necrosis and inflammation or number of infiltrating cells. Less fat tissue vs *dy*$^{W}$*/dy*$^{W}$ mice. | [79] |
| Mouse/ Mstn$^{-/-}$; scgd$^{-/-}$ (LGMD2F) | | | 36 weeks (exam.) | Increased | | | | | | | | | Hydroxyproline decreased vs scgd $^{-/-}$ | [5] |
| Mouse/ *Cav3*$^{P104L}$*;* *MSTN*$^{Pro}$ | | | 18 weeks | Increased | Increased weight in Triceps and Quad. Myofiber size increased vs caveolin-deficient mice | | Improved forearm grip-force | | | | | | | [45] |
| Mouse/dominant -negative myostatin | | | 16 weeks (exam.) | | Increased muscle weight of Gas., triceps, Quad. Increased CSA of Quad. | | | | | | | | | [80] |

Detailed results from previous studies as presented in Table 1. Changes are presented compared to controls of the particular study. The same reference may be found in different rows if various modalities of myostatin inhibition have been applied. References are listed according to reference list below. Abbreviations: AAV; adeno-associated virus, ActRIIB; activin receptor type IIB, CSA; cross-sectional area, CNF; centrally-nucleated fibers, EDL; m. extensor digitorum longus, eMHC; embryonic myosin heavy chain, Gas; m. gastrocnemius, GRMD; golden retriever muscular dystrophy i.m.; intra-muscular injection, i.p.; intraperitoneal injection, LGMD; limb-girdle muscular dystrophy, MHC; myosin heavy chain, OPMD; oculopharngeal muscular dystrophy Quad; m. quadriceps, s.c.; sub-cutaneous injection, SDH; succinate dehydrogenase, SMA; spinal muscle atrophy, Sol; m. soleus, TA; m. tibialis anterior.

Supplementary table 2: Detailed overview of published and unpublished clinical trials with myostatin inhibitors as per PubMed – U.S. National Library of Medicine and clinicaltrialsregister.eu and clinicaltrials.gov (access date February 23rd 2021).

| Treatment | Sponsor | Condition | Phase of trial | Age at treatment/ years | N | Length of treatment / regime | Primary outcome | Secondary outcome | Result | Status | Reference |
|---|---|---|---|---|---|---|---|---|---|---|---|
| **Neutralizing monoclonal antibodies** | | | | | | | | | | | |
| MYO-029 (Stamulumab) | Wyeth | Healthy subjects | I | N/A | 72 | MAD | Safety and tolerability, PK, PD | N/A | Well tolerated | Completed | NCT# 00563810 |
| | | BMD, FSHD, LGMD (2A, 2B, 2C, 2D, 2E, 2I) | I/II | >18 | 116 | 6 months | Safety and tolerability | Biological activity (manual muscle test, QMT, TFT, pulmonary function test, subject-reported outcome, MRI, change in muscle mass, LBM) | AE.<br>No effect in MMT, QMT, subject-reported outcome, LBM | Completed | [81]<br>EudraCT# 2004-000622-67<br>NCT# 00104078 |
| PF-06252616 (Domagrozumab) | Pfizer | Healthy subjects | I | 19-61 | 73 | Single and repeated administrations (3 weeks) | Safety and tolerability | PK/PD, total LBM | Well tolerated. Minor AE: Headache, fatigue, upper RTI, muscle spasms.<br>Single i.v: increased whole-body lean mass by 5,38 %,<br>Repeat i.v.: muscle vol increased 4,49 % | Completed | [82]<br>NCT# 01616277 |
| | | DMD | II | 6-16 | 121 | 96 weeks | Safety and tolerability, mean change 4 stair climb | TFT, pulmonary function tests, muscle volume, PK/PD | No significant between-group differences in any secondary clinical endpoints, withdrawn | Terminated | [83]<br>NCT# 02310763<br>Extension: NCT# 02907619 |
| | | LGMD2I (FKRP) | I/II | >18 | 19 | MAD, 32 weeks | Safety and tolerability | Muscle strength, TFTs, pulmonary function, LBM, PK, PD<br>Exploratory outcome: muscle fat fraction | Preliminary results on clinicaltrials.gov per February 23rd, 2021 | Completed | NCT#02841267 |
| LY2495655 (Landogrozumab) | Lilly | Healthy subjects | I | N/A | 64 | Single dose and 8 weeks multiple doses | "Clinically significant effect" | PK, PD, thigh muscle volume | Well tolerated. Thigh muscle volume increased | Completed | [84]<br>NCT# 01341470 |
| | | Advanced cancer | I | 71 | 29 | Dose-escalation for 8 weeks | Safety and tolerability | PK | Well tolerated. Increased muscle volume at two of administered doses. | Completed | [84]<br>NCT# 01524224 |
| | | Pancreatic Cancer /cachexia | II | 45-86 | 125 | 8 weeks, opportunity of extended treatment cycles of 4 weeks | Overall survival | Progression-free survival, tumor response, duration of response, LBM, TFT, PRO, pain | Terminated due to lack of effect and imbalance in death rates between treatment arms. | Completed /Terminated | [85]<br>NCT# 01505530 |
| | | Older, weak fallers | II | 75-99 | 201 | 20 weeks | Change in appendicular LBM | TFTs, gait speed, QMT, body composition, rate of falls, myostatin serum concentration | Appendicular LBM, TFTs, gait speed improved | Completed | [86]<br>NCT# 01604408 |
| | | Osteoarthritis undergoing total hip replacement | II | >50 | 400 | 12 weeks | Change in appendicular LBM | Secondary: QMT, PRO, whole-body-composition | Dose dependent increase in appendicular LBM and decreased fat, not enough to reach threshold of primary objective | Completed | [87]<br>NCT# 01369511 |
| REGN1033 (Trevogrumab) / SAR391786 | Regeneron/ Sanofi | Healthy subjects | I | >60 | 76, 60 | SAD | Assessment of safety, tolerability, administration | N/A | Results not reported (both studies) | Completed | NCT# 01507402, NCT# 01720576 |
| | | Healthy subjects | I | >60 | 125 | N/A | Change in total lean mass | Safety and tolerability, appendicular lean mass | Results not reported | Completed | NCT# 01910220 |
| | | Healthy subjects | I | 18-65 | 28 | Single dose | PK in two different formulations of drug | Safety and tolerability | Results not reported | Completed | NCT# 02741739 |
| | | Sarcopenia | II | >70 | 253 | 3 months | Change in total LBM | AE, appendicular lean mass, gait speed, SPPB, DXA-evaluated body composition, 6MWT, QMT, TFT | Results not reported | Completed | NCT# 01963598 |

| | | | | | | | | | | | |
|---|---|---|---|---|---|---|---|---|---|---|---|
| | | sIBM | II | 45-75 | N/A | N/A | Change in total lean mass | AE, TFT, 6MWT, 10MWT, QMT | Withdrawn | Withdrawn | NCT# 03710941 |
| REGN2477 (Garetosmab, Activin A-antibody) alone and in combination with REGN1033 | Regeneron | Healthy subjects | I | 35-70 | 82 | Ascending dose | Safety and tolerability | Thigh muscle volume, DXA-evaluated body composition, PK | Results not reported | Completed | NCT# 02943239 |
| SRK-015 (Apitegromab) | Scholar Rock | SMA 2, SMA 3 | II | 2-21 | 58 | 1 year | Change from Baseline in the Revised Hammersmith Scale or Hammersmith Functional Motor Scale Expanded (HFMSE) | N/A | Results not reported | Active per January 31., 2021 | NCT# 03921528 |
| GYM329 /RG6237 | Chugai Pharmaceutic al / Roche | Healthy subjects (limb immobilization) | I | 18-39 | 48 | 4 week parallel group design | Thight muscle strength | Safety and tolerability, PK, PD | Results not reported | Recruiting per January 31, 2021 | NCT# 04708847 |
| **Soluble ActRIIB** | | | | | | | | | | | |
| ACE-031 (Ramatercept) | Acceleron | Healthy subjects | Ia | 52-58 | 48 | SAD | Safety and tolerability | PK/PD, body mass evaluation by DXA and MRI | Well tolerated | Completed | [88] NCT# 00755638 |
| | | Healthy subjects | Ib | 45-75 | 70 | MAD, 3 months | Safety and tolerability | PK/PD | Adverse effect (epistaxis) Increased LBM and thigh muscle volume | Completed | NCT# 00952887 |
| | | DMD | II | 9-10 | 24, 11 in extensio n | 12 weeks | Safety and tolerability | PK/PD (MRI evaluation, bone mineral density, TFT)) | Body mass, Bone mineral density MD improved vs baseline (BL) No difference vs placebo<br><br>AE (telangiectasias, epistaxis) | Terminated | [89] NCT# 01099761 Extension: NCT# 01239758 |
| ACE-2494 | | Healthy subjects | I | 45-75 | 48 | SAD | Safety and tolerability | PK/PD, DXA-evaluated body composition, thigh muscle volume evaluated by MRI | Development of anti-drug antibodies | Terminated | [90] NCT# 03478319 |
| **Follistatin-Fc** | | | | | | | | | | | |
| ACE-083 | Acceleron | Healthy subjects | I | 45-73 | 58 | Single or double doses of intramuscular injections | Safety and tolerability | PK/PD, MRI/DXA evaluation, QMT | Well tolerated RF, TA increased volume Muscle strength no diff | Completed | [91] NCT# 02257489 |
| | | FSH | II | >18 | 58 | 6 months | Safety and tolerability | PK, PD, QMT, TFT, QOL | Did not meet secondary endpoints | Terminated | NCT# 02927080 |
| | | Charcot–Marie–Tooth | II | >18 | 42 | 3 months, 6 months vs placebo followed by 6 months open label | Safety and tolerability, change in muscle volume estimated by MRI | PK/PD, Muscular fat infiltration, QMT, TFT, QOL, CMT examination score) | Did not meet secondary endpoints | Terminated | NCT# 03124459 |
| **Antimyostatin adnectin** | | | | | | | | | | | |
| BMS-986089 | Bristol-Meyers-Squibb / Hoffmann-La Roche | Healthy subjects | I | 21-55 | 140 | SAD, MAD | Safety and tolerability | Pharmacokinetics | Results not reported | Completed | NCT# 02145234 |
| RG6202 / BMS-986089 / RO-7239361 | | DMD | Ib/II | 5-10 | 43 | MAD, 24 weeks | Safety and tolerability | Thigh contractive tissue, CSA, PK | No AE. Increased LBM | Terminated | NCT# 02515669 [92] |
| RO-7239361 | | DMD | II/III | 6-11 | 166 | 48 weeks | Changes in North Star Ambulatory Assessment score | TFT, QMT, 6MWT, walk, run and stride velocity | Primary endpoint not acheived | Discontinued | NCT# 03039686 [93] |
| **Anti-ActRIIB antibody** | | | | | | | | | | | |
| BYM-338 (Bimagrumab) | Novartis | sIBM | II | 45-78 | 14 | Single dose | Change in muscle volume | Body composition, LBM, QMT, TFT, 6MWT | Increased thigh muscle volume and LBM 8 weeks after treatment | Completed | [94] NCT# 01423110 Extension: NCT# 02250443 (terminated early) |

| | | | | | | | | | | | |
|---|---|---|---|---|---|---|---|---|---|---|---|
| | | | | | | | | | Increased 6MWT 16 weeks after treatment | | |
| | | sIBM | IIb/III | Mean 69 | 251 | 52 weeks | Change in 6MWT | LBM, QMT, sIBM functional assessment, rate of falls, SPPB | Increased LBM and improved sIBM function assessment. 6MWT not improved | Completed | [95,96] NCT# 01925209 EudraCT# 2013-000705-23 Extension: NCT# 02573467 EudraCT# 2015-001411-12) |
| | | Sarcopenia | II | 65-86 | 40 | Single infusion | Thigh muscle volume, intramuscular and subcutaneous fat tissue | Total LBM, QMT, TFT | Thigh muscle vol increased Walking speed increased in subgroup with slow walking speed | Completed | [97] NCT# 01601600 |
| | | Sarcopenia | II | >70 | 217 (Extension: 160) | 6 months | Change from baseline in SPPB | Safety, tolerability, 6MWT, gait speed, total LBM | Increased appendicular skeletal muscle index and LBM from baseline in 700 mg treatment cohort. No functional improvement in publication [98] | Completed | [98] NCT# 02333331 EudraCT# 2014-003482-25 Extension: NCT# 02468674 Extension: 2015-000471-27 |
| | | Patients undergoing surgical treatment of hip fracture | IIa / IIb | Not reported | 245 planned | Single infusion, 24 weeks follow up | Change in total LBM | Gait speed, SPPB, safety and tolerability, rate of falls | Results not reported | Completed | NCT# 02152761 EudraCT# 2013-003439-31 |
| | | Casting-induced muscle atrophy (healthy) | | 24 | 16 | Single administration | Thigh muscle volume, change in Intramuscular and subcutaneous adipose tissue | QMT, Safety and tolerability | Thigh muscle vol increased Strength no diff | | [99] No clinical trial ID specified in article |
| | | COPD | II | 63.7 | 67 | Two doses over 24 weeks period | Change in thigh muscle volume | 6MWT, PK | Thigh muscle volume increased. No difference in 6MWT | Completed | [100] NCT# 01669174 |
| | | Cancer cachexia (lung or pancreas) | II | Average 62 | 57 | Single dose, 8 weeks follow up | Change in thigh muscle volume | Body weight, PK/PD, bone mineral density, LBM, physical activity levels | P-value not calculated, appears like no effect of treatment | Completed | NCT# 01433263 |
| | | Type II diabetes | II | 18-75 | 68 | Monthly administrations, time frame not supplied | Change in body fat mass | HbA1c change, PK, body weight change, insulin resistance | Results not reported | Completed | NCT# 03005288 |
| **Follistatin gene therapy** | | | | | | | | | | | |
| AAV1.CMV.FS344 | Children's Hospital/ Milo Biotech | BMD | I/IIa (no placebo control) | 24-27 | 6 | Single treatment, 6 months of observation | Safety, change in 6MWT | QMT of quadriceps, muscle histology | 6MWT increased in four of six subjects, improved histological findings | Completed | [101] NCT# 01519349 |
| | | sIBM | I/IIa | Average 65,9 | 14 | Single treatment including exercise encouragement, followed for 1-2 years | 6MWT | TFT, Biopsy, western blotting) | Improvement in 6MWT vs controls, improved histological finding | Completed | [102] |
| rAAV1.CMV. huFollistatin344 | Jerry R. Mendell / Milo Therapeutics | DMD | I/II | N/A | 3 | Single dose, 24 months follow-up | AE | 6MWT, size of muscle fibers | Results not reported | Completed | NCT# 02354781 |
| **Antimyostatin peptibody** | | | | | | | | | | | |
| AMG-745 / PINTA 745 | Amgen | Prostate cancer in patients treated by androgen deprivation therapy | I | 71-73 | 54 | 4 weeks | AE, PK, DXA, QMT, SPPB, TFT | N/A | LBM increased, decreased fat mass. No effect on physical function or lower extremity strength | Completed | [103] |
| | | Age-associated muscle loss | II | N/A | N/A | N/A | Thigh CSA | QMT, TFT, 6MWT PK | Withdrawn | Withdrawn | NCT# 00975104 |
| | | End stage renal disease, kidney disease, | I/II | 18-85 | 51 | 12 weeks | Safety and tolerability, LBM change | LBM, appendicular lean mass, mid upper arm muscle circumference, TFT, 6MWT | No results posted | Completed | NCT# 01958970 |

| | | | | | | | | | | | |
|---|---|---|---|---|---|---|---|---|---|---|---|
| | | protein energy easting | | | | | | | | | |
| **Myostatin inhibition (information on myostatin inhibitory strategy not available)** | | | | | | | | | | | |
| BLS-M22 (oral capsule) | BioLeaders Corporation | Healthy subjects | I | 19-55 | 37 | 4-5 weeks | Safety and tolerability | PK, immunogenicity, changes in muscle mass | No results posted | Recruiting | NCT# 03789734 |

References are listed according to reference list below. Abbreviations: 10MWT; 10-minute walking test, 6MWT; 6-minute walking test, AE; Adverse events, BMD; Becker Muscular Dystrophy, COPD; chronic obstructive pulmonary disorder, CSA; Cross-sectional area, DMD; Duchenne Muscular Dystrophy, DXA; Dual-energy X-ray absorption, FSHD; Facio-scapulo-humoral dystrophy, LBM; Lean body mass, MRI; Magnetic resonance imaging, N/A; Not available, PRO; Patient reported outcome, PD; Pharmacodynamics, PK; Pharmacokinetics, QMT; Quantitative muscle testing, QOL; Quality of life, sIBM; spontaneous inclusion body myositis, SMA; spinal muscle atrophy, SPPB; Short Physical Performance Battery, TFT; Timed function test.

## References


1. Whittemore, L.-A.; Song, K.; Li, X.; Aghajanian, J.; Davies, M.; Girgenrath, S.; Hill, J.J.; Jalenak, M.; Kelley, P.; Knight, A.; et al. Inhibition of Myostatin in Adult Mice Increases Skeletal Muscle Mass and Strength. *Biochem. Biophys. Res. Commun.* **2003**, *300*, 965–971, doi:10.1016/S0006-291X(02)02953-4.
2. Camporez, J.-P.G.; Petersen, M.C.; Abudukadier, A.; Moreira, G.V.; Jurczak, M.J.; Friedman, G.; Haqq, C.M.; Petersen, K.F.; Shulman, G.I. Anti-Myostatin Antibody Increases Muscle Mass and Strength and Improves Insulin Sensitivity in Old Mice. *Proc. Natl. Acad. Sci. USA* **2016**, *113*, 2212–2217, doi:10.1073/pnas.1525795113.
3. Bogdanovich, S.; Krag, T.O.B.; Barton, E.R.; Morris, L.D.; Whittemore, L.-A.; Ahima, R.S.; Khurana, T.S. Functional Improvement of Dystrophic Muscle by Myostatin Blockade. *Nature* **2002**, *420*, 418–421, doi:10.1038/nature01154.
4. Bogdanovich, S.; McNally, E.M.; Khurana, T.S. Myostatin Blockade Improves Function but Not Histopathology in a Murine Model of Limb-Girdle Muscular Dystrophy 2C. *Muscle Nerve* **2008**, *37*, 308–316, doi:10.1002/mus.20920.
5. Parsons, S.A.; Millay, D.P.; Sargent, M.A.; McNally, E.M.; Molkentin, J.D. Age-Dependent Effect of Myostatin Blockade on Disease Severity in a Murine Model of Limb-Girdle Muscular Dystrophy. *Am. J. Pathol.* **2006**, *168*, 1975–1985, doi:10.2353/ajpath.2006.051316.
6. Murphy, K.T.; Ryall, J.G.; Snell, S.M.; Nair, L.; Koopman, R.; Krasney, P.A.; Ibebunjo, C.; Holden, K.S.; Loria, P.M.; Salatto, C.T.; et al. Antibody-Directed Myostatin Inhibition Improves Diaphragm Pathology in Young but Not Adult Dystrophic Mdx Mice. *Am. J. Pathol.* **2010**, *176*, 2425–2434, doi:10.2353/ajpath.2010.090932.
7. Girgenrath, S.; Song, K.; Whittemore, L.-A. Loss of Myostatin Expression Alters Fiber-Type Distribution and Expression of Myosin Heavy Chain Isoforms in Slow- and Fast-Type Skeletal Muscle. *Muscle Nerve* **2005**, *31*, 34–40, doi:10.1002/mus.20175.
8. Singh, P.; Rong, H.; Gordi, T.; Bosley, J.; Bhattacharya, I. Translational Pharmacokinetic/Pharmacodynamic Analysis of MYO-029 Antibody for Muscular Dystrophy. *Clin. Transl. Sci.* **2016**, *9*, 302–310, doi:10.1111/cts.12420.
9. Tinklenberg, J.A.; Siebers, E.M.; Beatka, M.J.; Meng, H.; Yang, L.; Zhang, Z.; Ross, J.A.; Ochala, J.; Morris, C.; Owens, J.M.; et al. Myostatin Inhibition Using MRK35 Produces Skeletal Muscle Growth and Tubular Aggregate Formation in Wild Type and TgACTA1D286G Nemaline Myopathy Mice. *Hum. Mol. Genet.* **2018**, *27*, 638–648, doi:10.1093/hmg/ddx431.
10. St. Andre, M.; Johnson, M.; Bansal, P.N.; Wellen, J.; Robertson, A.; Opsahl, A.; Burch, P.M.; Bialek, P.; Morris, C.; Owens, J. A Mouse Anti-Myostatin Antibody Increases Muscle Mass and Improves Muscle Strength and Contractility in the Mdx Mouse Model of Duchenne Muscular Dystrophy and Its Humanized Equivalent, Domagrozumab (PF-06252616), Increases Muscle Volume in Cynomolgus Monkeys. *Skelet. Muscle* **2017**, *7*, 25, doi:10.1186/s13395-017-0141-y.
11. Holzbaur, E.L.F.; Howland, D.S.; Weber, N.; Wallace, K.; She, Y.; Kwak, S.; Tchistiakova, L.A.; Murphy, E.; Hinson, J.; Karim, R.; et al. Myostatin Inhibition Slows Muscle Atrophy in Rodent Models of Amyotrophic Lateral Sclerosis. *Neurobiol. Dis.* **2006**, *23*, 697–707, doi:10.1016/j.nbd.2006.05.009.
12. Harish, P.; Malerba, A.; Lu-Nguyen, N.; Forrest, L.; Cappellari, O.; Roth, F.; Trollet, C.; Popplewell, L.; Dickson, G. Inhibition of Myostatin Improves Muscle Atrophy in Oculopharyngeal Muscular Dystrophy (OPMD). *J. Cachexia Sarcopenia Muscle* **2019**, *10*, 1016–1026, doi:10.1002/jcsm.12438.
13. Harish, P.; Forrest, L.; Herath, S.; Dickson, G.; Malerba, A.; Popplewell, L. Inhibition of Myostatin Reduces Collagen Deposition in a Mouse Model of Oculopharyngeal Muscular Dystrophy (OPMD) With Established Disease. *Front. Physiol.* **2020**, *11*, 184, doi:10.3389/fphys.2020.00184.
14. Latres, E.; Pangilinan, J.; Miloscio, L.; Bauerlein, R.; Na, E.; Potocky, T.B.; Huang, Y.; Eckersdorff, M.; Rafique, A.; Mastaitis, J.; et al. Myostatin Blockade with a Fully Human Monoclonal Antibody Induces Muscle Hypertrophy and Reverses Muscle Atrophy in Young and Aged Mice. *Skelet. Muscle* **2015**, *5*, doi:10.1186/s13395-015-0060-8.
15. Smith, R.C.; Cramer, M.S.; Mitchell, P.J.; Lucchesi, J.; Ortega, A.M.; Livingston, E.W.; Ballard, D.; Zhang, L.; Hanson, J.; Barton, K.; et al. Inhibition of Myostatin Prevents Microgravity-Induced Loss of Skeletal Muscle Mass and Strength. *PLoS ONE* **2020**, *15*, doi:10.1371/journal.pone.0230818.
16. Pirruccello-Straub, M.; Jackson, J.; Wawersik, S.; Webster, M.T.; Salta, L.; Long, K.; McConaughy, W.; Capili, A.; Boston, C.; Carven, G.J.; et al. Blocking Extracellular Activation of Myostatin as a Strategy for Treating Muscle Wasting. *Sci. Rep.* **2018**, *8*, doi:10.1038/s41598-018-20524-9.
17. Long, K.K.; O'Shea, K.M.; Khairallah, R.J.; Howell, K.; Paushkin, S.; Chen, K.S.; Cote, S.M.; Webster, M.T.; Stains, J.P.; Treece, E.; et al. Specific Inhibition of Myostatin Activation Is Beneficial in Mouse Models of SMA Therapy. *Hum. Mol. Genet.* **2019**, *28*, 1076–1089, doi:10.1093/hmg/ddy382.
18. Muramatsu, H.; Kuramochi, T.; Katada, H.; Ueyama, A.; Ruike, Y.; Ohmine, K.; Shida-Kawazoe, M.; Miyano-Nishizawa, R.; Shimizu, Y.; Okuda, M.; et al. Novel Myostatin-Specific Antibody Enhances Muscle Strength in Muscle Disease Models. *Sci. Rep.* **2021**, *11*, 2160, doi:10.1038/s41598-021-81669-8.

19. Lach-Trifilieff, E.; Minetti, G.C.; Sheppard, K.; Ibebunjo, C.; Feige, J.N.; Hartmann, S.; Brachat, S.; Rivet, H.; Koelbing, C.; Morvan, F.; et al. An Antibody Blocking Activin Type II Receptors Induces Strong Skeletal Muscle Hypertrophy and Protects from Atrophy. *Mol. Cell. Biol.* **2014**, *34*, 606–618, doi:10.1128/MCB.01307-13.
20. Bogdanovich, S.; Perkins, K.J.; Krag, T.O.B.; Whittemore, L.-A.; Khurana, T.S. Myostatin Propeptide-Mediated Amelioration of Dystrophic Pathophysiology. *FASEB J.* **2005**, *19*, 543–549, doi:10.1096/fj.04-2796com.
21. Qiao, C.; Li, J.; Jiang, J.; Zhu, X.; Wang, B.; Li, J.; Xiao, X. Myostatin Propeptide Gene Delivery by Adeno-Associated Virus Serotype 8 Vectors Enhances Muscle Growth and Ameliorates Dystrophic Phenotypes in *Mdx* Mice. *Hum. Gene Ther.* **2008**, *19*, 241–254, doi:10.1089/hum.2007.159.
22. Bartoli, M.; Poupiot, J.; Vulin, A.; Fougerousse, F.; Arandel, L.; Daniele, N.; Roudaut, C.; Noulet, F.; Garcia, L.; Danos, O.; et al. AAV-Mediated Delivery of a Mutated Myostatin Propeptide Ameliorates Calpain 3 but Not $\alpha$ -Sarcoglycan Deficiency. *Gene Ther.* **2007**, *14*, 733–740, doi:10.1038/sj.gt.3302928.
23. Pistilli, E.E.; Bogdanovich, S.; Goncalves, M.D.; Ahima, R.S.; Lachey, J.; Seehra, J.; Khurana, T. Targeting the Activin Type IIB Receptor to Improve Muscle Mass and Function in the Mdx Mouse Model of Duchenne Muscular Dystrophy. *Am. J. Pathol.* **2011**, *178*, 1287–1297, doi:10.1016/j.ajpath.2010.11.071.
24. Carlson, C.G.; Bruemmer, K.; Sesti, J.; Stefanski, C.; Curtis, H.; Ucran, J.; Lachey, J.; Seehra, J.S. Soluble Activin Receptor Type IIB Increased Forward Pulling Tension in the Mdx Mouse. *Muscle Nerve* **2011**, *43*, 694–699, doi:10.1002/mus.21944.
25. Akpan, I.; Goncalves, M.D.; Dhir, R.; Yin, X.; Pistilli, E.E.; Bogdanovich, S.; Khurana, T.S.; Ucran, J.; Lachey, J.; Ahima, R.S. The Effects of a Soluble Activin Type IIB Receptor on Obesity and Insulin Sensitivity. *Int. J. Obes.* **2009**, *33*, 1265–1273, doi:10.1038/ijo.2009.162.
26. Nagy, J.A.; Kapur, K.; Taylor, R.S.; Sanchez, B.; Rutkove, S.B. Electrical Impedance Myography as a Biomarker of Myostatin Inhibition with ActRIIB-MFc: A Study in Wild-Type Mice. *Future Sci. OA* **2018**, *4*, FSO308, doi:10.4155/fsoa-2018-0002.
27. Béchir, N.; Pecchi, É.; Relizani, K.; Vilmen, C.; Le Fur, Y.; Bernard, M.; Amthor, H.; Bendahan, D.; Giannesini, B. Mitochondrial Impairment Induced by Postnatal ActRIIB Blockade Does Not Alter Function and Energy Status in Exercising Mouse Glycolytic Muscle in Vivo. *Am. J. Physiol.-Endocrinol. Metab.* **2016**, *310*, E539–E549, doi:10.1152/ajpendo.00370.2015.
28. Tinklenberg, J.; Meng, H.; Yang, L.; Liu, F.; Hoffmann, R.G.; Dasgupta, M.; Allen, K.P.; Beggs, A.H.; Hardeman, E.C.; Pearsall, R.S.; et al. Treatment with ActRIIB-MFc Produces Myofiber Growth and Improves Lifespan in the Acta1 H40Y Murine Model of Nemaline Myopathy. *Am. J. Pathol.* **2016**, *186*, 1568–1581, doi:10.1016/j.ajpath.2016.02.008.
29. Tinklenberg, J.A.; Siebers, E.M.; Beatka, M.J.; Fickau, B.A.; Ayres, S.; Meng, H.; Yang, L.; Simpson, P.; Granzier, H.L.; Lawlor, M.W. Myostatin Inhibition Using ActRIIB-MFc Does Not Produce Weight Gain or Strength in the Nebulin Conditional KO Mouse. *J. Neuropathol. Exp. Neurol.* **2019**, *78*, 130–139, doi:10.1093/jnen/nly120.
30. Lawlor, M.W.; Read, B.P.; Edelstein, R.; Yang, N.; Pierson, C.R.; Stein, M.J.; Wermer-Colan, A.; Buj-Bello, A.; Lachey, J.L.; Seehra, J.S.; et al. Inhibition of Activin Receptor Type IIB Increases Strength and Lifespan in Myotubularin-Deficient Mice. *Am. J. Pathol.* **2011**, *178*, 784–793, doi:10.1016/j.ajpath.2010.10.035.
31. Lawlor, M.W.; Viola, M.G.; Meng, H.; Edelstein, R.V.; Liu, F.; Yan, K.; Luna, E.J.; Lerch-Gaggl, A.; Hoffmann, R.G.; Pierson, C.R.; et al. Differential Muscle Hypertrophy Is Associated with Satellite Cell Numbers and Akt Pathway Activation Following Activin Type IIB Receptor Inhibition in Mtm1 p.R69C Mice. *Am. J. Pathol.* **2014**, *184*, 1831–1842, doi:10.1016/j.ajpath.2014.03.003.
32. Relizani, K.; Mouisel, E.; Giannesini, B.; Hourdé, C.; Patel, K.; Gonzalez, S.M.; Jülich, K.; Vignaud, A.; Piétri-Rouxel, F.; Fortin, D.; et al. Blockade of ActRIIB Signaling Triggers Muscle Fatigability and Metabolic Myopathy. *Mol. Ther.* **2014**, *22*, 1423–1433, doi:10.1038/mt.2014.90.
33. Béchir, N.; Pecchi, E.; Vilmen, C.; Le Fur, Y.; Amthor, H.; Bernard, M.; Bendahan, D.; Giannesini, B. ActRIIB Blockade Increases Force-Generating Capacity and Preserves Energy Supply in Exercising Mdx Mouse Muscle in Vivo. *FASEB J.* **2016**, *30*, 3551–3562, doi:10.1096/fj.201600271RR.
34. Cadena, S.M.; Tomkinson, K.N.; Monnell, T.E.; Spaits, M.S.; Kumar, R.; Underwood, K.W.; Pearsall, R.S.; Lachey, J.L. Administration of a Soluble Activin Type IIB Receptor Promotes Skeletal Muscle Growth Independent of Fiber Type. *J. Appl. Physiol.* **2010**, *109*, 635–642, doi:10.1152/japplphysiol.00866.2009.
35. Béchir, N.; Pecchi, É.; Vilmen, C.; Bernard, M.; Bendahan, D.; Giannesini, B. Activin Type IIB Receptor Blockade Does Not Limit Adenosine Triphosphate Supply in Mouse Skeletal Muscle in Vivo. *Muscle Nerve* **2018**, *58*, 834–842, doi:10.1002/mus.26306.
36. Castonguay, R.; Lachey, J.; Wallner, S.; Strand, J.; Liharska, K.; Watanabe, A.E.; Cannell, M.; Davies, M.V.; Sako, D.; Troy, M.E.; et al. Follistatin-288-Fc Fusion Protein Promotes Localized Growth of Skeletal Muscle. *J. Pharmacol. Exp. Ther.* **2018**, jpet.118.252304, doi:10.1124/jpet.118.252304.
37. Hoogaars, W.M.H.; Mouisel, E.; Pasternack, A.; Hulmi, J.J.; Relizani, K.; Schuelke, M.; Schirwis, E.; Garcia, L.; Ritvos, O.; Ferry, A.; et al. Combined Effect of AAV-U7-Induced Dystrophin Exon Skipping and Soluble Activin Type IIB Receptor in Mdx Mice. *Hum. Gene Ther.* **2012**, *23*, 1269–1279, doi:10.1089/hum.2012.056.

38. Li, Z.B.; Zhang, J.; Wagner, K.R. Inhibition of Myostatin Reverses Muscle Fibrosis through Apoptosis. *J Cell Sci* **2012**, *125*, 3957–3965, doi:10.1242/jcs.090365.
39. Bondulich, M.K.; Jolinon, N.; Osborne, G.F.; Smith, E.J.; Rattray, I.; Neueder, A.; Sathasivam, K.; Ahmed, M.; Ali, N.; Benjamin, A.C.; et al. Myostatin Inhibition Prevents Skeletal Muscle Pathophysiology in Huntington's Disease Mice. *Sci. Rep.* **2017**, *7*, 14275, doi:10.1038/s41598-017-14290-3.
40. Pistilli, E.E.; Bogdanovich, S.; Mosqueira, M.; Lachey, J.; Seehra, J.; Khurana, T.S. Pretreatment with a Soluble Activin Type IIB Receptor/Fc Fusion Protein Improves Hypoxia-Induced Muscle Dysfunction. *Am. J. Physiol.-Regul. Integr. Comp. Physiol.* **2010**, *298*, R96–R103, doi:10.1152/ajpregu.00138.2009.
41. Chiu, C.-S.; Peekhaus, N.; Weber, H.; Adamski, S.; Murray, E.M.; Zhang, H.Z.; Zhao, J.Z.; Ernst, R.; Lineberger, J.; Huang, L.; et al. Increased Muscle Force Production and Bone Mineral Density in ActRIIB-Fc-Treated Mature Rodents. *J. Gerontol. A. Biol. Sci. Med. Sci.* **2013**, *68*, 1181–1192, doi:10.1093/gerona/glt030.
42. Hulmi, J.J.; Oliveira, B.M.; Silvennoinen, M.; Hoogaars, W.M.H.; Pasternack, A.; Kainulainen, H.; Ritvos, O. Exercise Restores Decreased Physical Activity Levels and Increases Markers of Autophagy and Oxidative Capacity in Myostatin/Activin-Blocked Mdx Mice. *Am. J. Physiol.-Endocrinol. Metab.* **2013**, *305*, E171–E182, doi:10.1152/ajpendo.00065.2013.
43. Lee, Y.-S.; Lehar, A.; Sebald, S.; Liu, M.; Swaggart, K.A.; Talbot, C.C.; Pytel, P.; Barton, E.R.; McNally, E.M.; Lee, S.-J. Muscle Hypertrophy Induced by Myostatin Inhibition Accelerates Degeneration in Dysferlinopathy. *Hum. Mol. Genet.* **2015**, *24*, 5711–5719, doi:10.1093/hmg/ddv288.
44. Iskenderian, A.; Liu, N.; Deng, Q.; Huang, Y.; Shen, C.; Palmieri, K.; Crooker, R.; Lundberg, D.; Kastrapeli, N.; Pescatore, B.; et al. Myostatin and Activin Blockade by Engineered Follistatin Results in Hypertrophy and Improves Dystrophic Pathology in Mdx Mouse More than Myostatin Blockade Alone. *Skelet. Muscle* **2018**, *8*, 34, doi:10.1186/s13395-018-0180-z.
45. Ohsawa, Y.; Hagiwara, H.; Nakatani, M.; Yasue, A.; Moriyama, K.; Murakami, T.; Tsuchida, K.; Noji, S.; Sunada, Y. Muscular Atrophy of Caveolin-3–Deficient Mice Is Rescued by Myostatin Inhibition. *J. Clin. Invest.* **2006**, *116*, 2924–2934, doi:10.1172/JCI28520.
46. Tauer, J.T.; Rauch, F. Novel ActRIIB Ligand Trap Increases Muscle Mass and Improves Bone Geometry in a Mouse Model of Severe Osteogenesis Imperfecta. *Bone* **2019**, *128*, 115036, doi:10.1016/j.bone.2019.115036.
47. Sumner, C.J.; Wee, C.D.; Warsing, L.C.; Choe, D.W.; Ng, A.S.; Lutz, C.; Wagner, K.R. Inhibition of Myostatin Does Not Ameliorate Disease Features of Severe Spinal Muscular Atrophy Mice. *Hum. Mol. Genet.* **2009**, *18*, 3145–3152, doi:10.1093/hmg/ddp253.
48. Morvan, F.; Rondeau, J.-M.; Zou, C.; Minetti, G.; Scheufler, C.; Scharenberg, M.; Jacobi, C.; Brebbia, P.; Ritter, V.; Toussaint, G.; et al. Blockade of Activin Type II Receptors with a Dual Anti-ActRIIA/IIB Antibody Is Critical to Promote Maximal Skeletal Muscle Hypertrophy. *Proc. Natl. Acad. Sci. USA.* **2017**, *114*, 12448–12453, doi:10.1073/pnas.1707925114.
49. Haidet, A.M.; Rizo, L.; Handy, C.; Umapathi, P.; Eagle, A.; Shilling, C.; Boue, D.; Martin, P.T.; Sahenk, Z.; Mendell, J.R.; et al. Long-Term Enhancement of Skeletal Muscle Mass and Strength by Single Gene Administration of Myostatin Inhibitors. *Proc. Natl. Acad. Sci. USA.* **2008**, *105*, 4318–4322, doi:10.1073/pnas.0709144105.
50. Kota, J.; Handy, C.R.; Haidet, A.M.; Montgomery, C.L.; Eagle, A.; Rodino-Klapac, L.R.; Tucker, D.; Shilling, C.J.; Therlfall, W.R.; Walker, C.M.; et al. Follistatin Gene Delivery Enhances Muscle Growth and Strength in Nonhuman Primates. *Sci. Transl. Med.* **2009**, *1*, 6ra15, doi:10.1126/scitranslmed.3000112.
51. Pearsall, R.S.; Davies, M.V.; Cannell, M.; Li, J.; Widrick, J.; Mulivor, A.W.; Wallner, S.; Troy, M.E.; Spaits, M.; Liharska, K.; et al. Follistatin-Based Ligand Trap ACE-083 Induces Localized Hypertrophy of Skeletal Muscle with Functional Improvement in Models of Neuromuscular Disease. *Sci. Rep.* **2019**, *9*, 1–14, doi:10.1038/s41598-019-47818-w.
52. Rose, F.F.; Mattis, V.B.; Rindt, H.; Lorson, C.L. Delivery of Recombinant Follistatin Lessens Disease Severity in a Mouse Model of Spinal Muscular Atrophy. *Hum. Mol. Genet.* **2009**, *18*, 997–1005, doi:10.1093/hmg/ddn426.
53. Nakatani, M.; Takehara, Y.; Sugino, H.; Matsumoto, M.; Hashimoto, O.; Hasegawa, Y.; Murakami, T.; Uezumi, A.; Takeda, S.; Noji, S.; et al. Transgenic Expression of a Myostatin Inhibitor Derived from Follistatin Increases Skeletal Muscle Mass and Ameliorates Dystrophic Pathology in *Mdx* Mice. *FASEB J.* **2008**, *22*, 477–487, doi:10.1096/fj.07-8673com.
54. Miller, T.M.; Kim, S.H.; Yamanaka, K.; Hester, M.; Umapathi, P.; Arnson, H.; Rizo, L.; Mendell, J.R.; Gage, F.H.; Cleveland, D.W.; et al. Gene Transfer Demonstrates That Muscle Is Not a Primary Target for Non-Cell-Autonomous Toxicity in Familial Amyotrophic Lateral Sclerosis. *Proc. Natl. Acad. Sci. USA* **2006**, *103*, 19546–19551, doi:10.1073/pnas.0609411103.
55. Foster, K.; Graham, I.R.; Otto, A.; Foster, H.; Trollet, C.; Yaworsky, P.J.; Walsh, F.S.; Bickham, D.; Curtin, N.A.; Kawar, S.L.; et al. Adeno-Associated Virus-8-Mediated Intravenous Transfer of Myostatin Propeptide Leads to Systemic Functional Improvements of Slow but Not Fast Muscle. *Rejuvenation Res.* **2009**, *12*, 85–94, doi:10.1089/rej.2008.0815.

56. Liu, M.; Hammers, D.W.; Barton, E.R.; Sweeney, H.L. Activin Receptor Type IIB Inhibition Improves Muscle Phenotype and Function in a Mouse Model of Spinal Muscular Atrophy. *PLoS ONE* **2016**, *11*, e0166803, doi:10.1371/journal.pone.0166803.
57. Morine, K.J.; Bish, L.T.; Selsby, J.T.; Gazzara, J.A.; Pendrak, K.; Sleeper, M.M.; Barton, E.R.; Lee, S.-J.; Sweeney, H.L. Activin IIB Receptor Blockade Attenuates Dystrophic Pathology in a Mouse Model of Duchenne Muscular Dystrophy. *Muscle Nerve* **2010**, *42*, 722–730, doi:10.1002/mus.21743.
58. Hammers, D.W.; Hart, C.C.; Patsalos, A.; Matheny, M.K.; Wright, L.A.; Nagy, L.; Sweeney, H.L. Glucocorticoids Counteract Hypertrophic Effects of Myostatin Inhibition in Dystrophic Muscle. *JCI Insight* **2020**, *5*, doi:10.1172/jci.insight.133276.
59. Morine, K.J.; Bish, L.T.; Pendrak, K.; Sleeper, M.M.; Barton, E.R.; Sweeney, H.L. Systemic Myostatin Inhibition via Liver-Targeted Gene Transfer in Normal and Dystrophic Mice. *PLoS ONE* **2010**, *5*, e9176, doi:10.1371/journal.pone.0009176.
60. Zhou, H.; Meng, J.; Malerba, A.; Catapano, F.; Sintusek, P.; Jarmin, S.; Feng, L.; Lu-Nguyen, N.; Sun, L.; Mariot, V.; et al. Myostatin Inhibition in Combination with Antisense Oligonucleotide Therapy Improves Outcomes in Spinal Muscular Atrophy. *J. Cachexia Sarcopenia Muscle* **2020**, *11*, 768–782, doi:10.1002/jcsm.12542.
61. Bish, L.T.; Sleeper, M.M.; Forbes, S.C.; Morine, K.J.; Reynolds, C.; Singletary, G.E.; Trafny, D.; Pham, J.; Bogan, J.; Kornegay, J.N.; et al. Long-Term Systemic Myostatin Inhibition via Liver-Targeted Gene Transfer in Golden Retriever Muscular Dystrophy. *Hum. Gene Ther.* **2011**, *22*, 1499–1509, doi:10.1089/hum.2011.102.
62. Monestier, O.; Brun, C.; Heu, K.; Passet, B.; Malhouroux, M.; Magnol, L.; Vilotte, J.-L.; Blanquet, V. Ubiquitous Gasp1 Overexpression in Mice Leads Mainly to a Hypermuscular Phenotype. *BMC Genomics* **2012**, *13*, 541, doi:10.1186/1471-2164-13-541.
63. Malerba, A.; Kang, J.K.; McClorey, G.; Saleh, A.F.; Popplewell, L.; Gait, M.J.; Wood, M.J.; Dickson, G. Dual Myostatin and Dystrophin Exon Skipping by Morpholino Nucleic Acid Oligomers Conjugated to a Cell-Penetrating Peptide Is a Promising Therapeutic Strategy for the Treatment of Duchenne Muscular Dystrophy. *Mol. Ther. Nucleic Acids* **2012**, *1*, e62, doi:10.1038/mtna.2012.54.
64. Lu-Nguyen, N.; Malerba, A.; Popplewell, L.; Schnell, F.; Hanson, G.; Dickson, G. Systemic Antisense Therapeutics for Dystrophin and Myostatin Exon Splice Modulation Improve Muscle Pathology of Adult Mdx Mice. *Mol. Ther. Nucleic Acids* **2017**, *6*, 15–28, doi:10.1016/j.omtn.2016.11.009.
65. Kang, J.K.; Malerba, A.; Popplewell, L.; Foster, K.; Dickson, G. Antisense-Induced Myostatin Exon Skipping Leads to Muscle Hypertrophy in Mice Following Octa Guanidine Morpholino Oligomer Treatment. *Mol. Ther.* **2011**, *19*, 159–164, doi:10.1038/mt.2010.212.
66. Dumonceaux, J.; Marie, S.; Beley, C.; Trollet, C.; Vignaud, A.; Ferry, A.; Butler-Browne, G.; Garcia, L. Combination of Myostatin Pathway Interference and Dystrophin Rescue Enhances Tetanic and Specific Force in Dystrophic Mdx Mice. *Mol. Ther.* **2010**, *18*, 881–887, doi:10.1038/mt.2009.322.
67. Weng, S.; Gao, F.; Wang, J.; Li, X.; Chu, B.; Wang, J.; Yang, G. Improvement of Muscular Atrophy by AAV-SaCas9-Mediated Myostatin Gene Editing in Aged Mice. *Cancer Gene Ther.* **2020**, *27*, 960–975, doi:10.1038/s41417-020-0178-7.
68. McPherron, A.C.; Lawler, A.M.; Lee, S.-J. Regulation of Skeletal Muscle Mass in Mice by a New TGF-p Superfamily Member. *Nature* **1997**, *387*, 83–90, doi:10.1038/387083a0.
69. Mendias, C.L.; Marcin, J.E.; Calerdon, D.R.; Faulkner, J.A. Contractile Properties of EDL and Soleus Muscles of Myostatin-Deficient Mice. *J. Appl. Physiol.* **2006**, *101*, 898–905, doi:10.1152/japplphysiol.00126.2006.
70. Amthor, H.; Macharia, R.; Navarrete, R.; Schuelke, M.; Brown, S.C.; Otto, A.; Voit, T.; Muntoni, F.; Vrbóva, G.; Partridge, T.; et al. Lack of Myostatin Results in Excessive Muscle Growth but Impaired Force Generation. *Proc. Natl. Acad. Sci.* **2007**, *104*, 1835–1840, doi:10.1073/pnas.0604893104.
71. Matsakas, A.; Mouisel, E.; Amthor, H.; Patel, K. Myostatin Knockout Mice Increase Oxidative Muscle Phenotype as an Adaptive Response to Exercise. *J. Muscle Res. Cell Motil.* **2010**, *31*, 111–125, doi:10.1007/s10974-010-9214-9.
72. Hennebry, A.; Berry, C.; Siriett, V.; O'Callaghan, P.; Chau, L.; Watson, T.; Sharma, M.; Kambadur, R. Myostatin Regulates Fiber-Type Composition of Skeletal Muscle by Regulating MEF2 and MyoD Gene Expression. *Am. J. Physiol.-Cell Physiol.* **2009**, *296*, C525–C534, doi:10.1152/ajpcell.00259.2007.
73. Hennebry, A.; Oldham, J.; Shavlakadze, T.; Grounds, M.D.; Sheard, P.; Fiorotto, M.L.; Falconer, S.; Smith, H.K.; Berry, C.; Jeanplong, F.; et al. IGF1 Stimulates Greater Muscle Hypertrophy in the Absence of Myostatin in Male Mice. *J. Endocrinol.* **2017**, *234*, 187–200, doi:10.1530/JOE-17-0032.
74. Qaisar, R.; Renaud, G.; Morine, K.; Barton, E.R.; Sweeney, H.L.; Larsson, L. Is Functional Hypertrophy and Specific Force Coupled with the Addition of Myonuclei at the Single Muscle Fiber Level? *FASEB J.* **2011**, *26*, 1077–1085, doi:10.1096/fj.11-192195.
75. Kocsis, T.; Trencsenyi, G.; Szabo, K.; Baan, J.A.; Muller, G.; Mendler, L.; Garai, I.; Reinauer, H.; Deak, F.; Dux, L.; et al. Myostatin Propeptide Mutation of the Hypermuscular Compact Mice Decreases the Formation of Myostatin and Improves Insulin Sensitivity. *Am. J. Physiol.-Endocrinol. Metab.* **2016**, *312*, E150–E160, doi:10.1152/ajpendo.00216.2016.

76. Baán, J.A.; Kocsis, T.; Keller-Pintér, A.; Müller, G.; Zádor, E.; Dux, L.; Mendler, L. The Compact Mutation of Myostatin Causes a Glycolytic Shift in the Phenotype of Fast Skeletal Muscles. *J. Histochem. Cytochem.* **2013**, *61*, 889–900, doi:10.1369/0022155413503661.
77. Wagner, K.R.; McPherron, A.C.; Winik, N.; Lee, S.-J. Loss of Myostatin Attenuates Severity of Muscular Dystrophy in Mdx Mice. *Ann. Neurol.* **2002**, *52*, 832–836, doi:10.1002/ana.10385.
78. Wagner, K.R.; Liu, X.; Chang, X.; Allen, R.E. Muscle Regeneration in the Prolonged Absence of Myostatin. *Proc. Natl. Acad. Sci. USA.* **2005**, *102*, 2519–2524, doi:10.1073/pnas.0408729102.
79. Li, Z.; Shelton, G.D.; Engvall, E. Elimination of Myostatin Does Not Combat Muscular Dystrophy in Dy Mice but Increases Postnatal Lethality. *Am. J. Pathol.* **2005**, *166*, 491–497.
80. Zhu, X.; Hadhazy, M.; Wehling, M.; Tidball, J.G.; McNally, E.M. Dominant Negative Myostatin Produces Hypertrophy without Hyperplasia in Muscle. *FEBS Lett.* **2000**, *474*, 71–75, doi:10.1016/S0014-5793(00)01570-2.
81. Wagner, K.R.; Fleckenstein, J.L.; Amato, A.A.; Barohn, R.J.; Bushby, K.; Escolar, D.M.; Flanigan, K.M.; Pestronk, A.; Tawil, R.; Wolfe, G.I.; et al. A Phase I/IItrial of MYO-029 in Adult Subjects with Muscular Dystrophy. *Ann. Neurol.* **2008**, *63*, 561–571, doi:10.1002/ana.21338.
82. Bhattacharya, I.; Pawlak, S.; Marraffino, S.; Christensen, J.; Sherlock, S.P.; Alvey, C.; Morris, C.; Arkin, S.; Binks, M.; Safety, Tolerability, Pharmacokinetics, and Pharmacodynamics of Domagrozumab (PF-06252616), an Antimyostatin Monoclonal Antibody, in Healthy Subjects. *Clin. Pharmacol. Drug Dev.* **2018**, *7*, 484–497, doi:10.1002/cpdd.386.
83. Wagner, K.R.; Abdel-Hamid, H.Z.; Mah, J.K.; Campbell, C.; Guglieri, M.; Muntoni, F.; Takeshima, Y.; McDonald, C.M.; Kostera-Pruszczyk, A.; Karachunski, P.; et al. Randomized Phase 2 Trial and Open-Label Extension of Domagrozumab in Duchenne Muscular Dystrophy. *Neuromuscul. Disord. NMD* **2020**, *30*, 492–502, doi:10.1016/j.nmd.2020.05.002.
84. Jameson, G.S.; Von Hoff, D.D.; Weiss, G.J.; Richards, D.A.; Smith, D.A.; Becerra, C.; Benson, M.C.; Yuan, Z.; Robins, D.A.; Turik, M.; et al. Safety of the Antimyostatin Monoclonal Antibody LY2495655 in Healthy Subjects and Patients with Advanced Cancer. *J. Clin. Oncol.* **2012**, *30*, 2516–2516, doi:10.1200/jco.2012.30.15_suppl.2516.
85. Golan, T.; Geva, R.; Richards, D.; Madhusudan, S.; Lin, B.K.; Wang, H.T.; Walgren, R.A.; Stemmer, S.M. LY2495655, an Antimyostatin Antibody, in Pancreatic Cancer: A Randomized, Phase 2 Trial: LY2495655 in Patients with Stage II-IV Pancreatic Cancer. *J. Cachexia Sarcopenia Muscle* **2018**, *9*, 871–879, doi:10.1002/jcsm.12331.
86. Becker, C.; Lord, S.R.; Studenski, S.A.; Warden, S.J.; Fielding, R.A.; Recknor, C.P.; Hochberg, M.C.; Ferrari, S.L.; Blain, H.; Binder, E.F.; et al. Myostatin Antibody (LY2495655) in Older Weak Fallers: A Proof-of-Concept, Randomised, Phase 2 Trial. *Lancet Diabetes Endocrinol.* **2015**, *3*, 948–957, doi:10.1016/S2213-8587(15)00298-3.
87. Woodhouse, L.; Gandhi, R.; Warden, S.J.; Poiraudeau, S.; Myers, S.L.; Benson, C.T.; Hu, L.; Ahmad, Q.I.; Linnemeier, P.; Gomez, E.V.; et al. A Phase 2 Randomized Study Investigating the Efficacy and Safety of Myostatin Antibody LY2495655 versus Placebo in Patients Undergoing Elective Total Hip Arthroplasty. *J. Frailty Aging* **2016**, *5*, 62–70, doi:10.14283/jfa.2016.81.
88. Attie, K.M.; Borgstein, N.G.; Yang, Y.; Condon, C.H.; Wilson, D.M.; Pearsall, A.E.; Kumar, R.; Willins, D.A.; Seehra, J.S.; Sherman, M.L. A Single Ascending-Dose Study of Muscle Regulator Ace-031 in Healthy Volunteers. *Muscle Nerve* **2013**, *47*, 416–423, doi:10.1002/mus.23539.
89. Campbell, C.; McMillan, H.J.; Mah, J.K.; Tarnopolsky, M.; Selby, K.; McClure, T.; Wilson, D.M.; Sherman, M.L.; Escolar, D.; Attie, K.M. Myostatin Inhibitor ACE-031 Treatment of Ambulatory Boys with Duchenne Muscular Dystrophy: Results of a Randomized, Placebo-Controlled Clinical Trial. *Muscle Nerve* **2017**, *55*, 458–464, doi:10.1002/mus.25268.
90. Acceleron Discontinues Development of Phase 1 Molecule ACE-2494 (April 4, 2019) Available online: http://investor.acceleronpharma.com/news-releases/news-release-details/acceleron-discontinues-development-phase-1-molecule-ace-2494 (accessed on 20 October 2019).
91. Glasser, C.E.; Gartner, M.R.; Wilson, D.; Miller, B.; Sherman, M.L.; Attie, K.M. Locally Acting ACE-083 Increases Muscle Volume in Healthy Volunteers. *Muscle Nerve* **2018**, *57*, 921–926, doi:10.1002/mus.26113.
92. Wagner, K.R.; Wong, B.L.; Byrne, B.J.; Tian, C.; Jacobsen, L.K.; Tirucherai, G.S.; Rabbia, M.; Kletzl, H.; Dukart, J.; Ong, R.; et al. A Phase 1b/2 Study of the Anti-Myostatin Adnectin RG6206 (BMS-986089) in Ambulatory Boys with Duchenne Muscular Dystrophy: A 72-Week Treatment Update (P1.6-062). *Neurology* **2018**, *92*, P1.6–062.
93. Roche/Genentech Announces Decision to Discontinue Development of RG6206 (RO7239361) Available online: https://www.parentprojectmd.org/roche-genentech-announces-decision-to-discontinue-development-of-rg6206-ro7239361/ (accessed on 13 January 2021).
94. Amato, A.A.; Sivakumar, K.; Goyal, N.; David, W.S.; Salajegheh, M.; Praestgaard, J.; Lach-Trifilieff, E.; Trendelenburg, A.-U.; Laurent, D.; Glass, D.J.; et al. Treatment of Sporadic Inclusion Body Myositis with Bimagrumab. *Neurology* **2014**, *83*, 2239–2246, doi:10.1212/WNL.0000000000001070.
95. Amato, A.A.; Auberson, L.Z.; Hanna, M.G.; Badrising, U.A.; Needham, M.; Chinoy, H.; Aoki, M.; Koumaras, B.; Tanko, L.; Wu, M.; et al. Long-Term Efficacy and Safety of Bimagrumab in Inclusion Body Myositis: 2 Years Results (S38.003). *Neurology* **2018**, *90*, S38.003.

96. Amato, A.A.; Hanna, M.G.; Machado, P.M.; Badrising, U.A.; Chinoy, H.; Benveniste, O.; Karanam, A.K.; Wu, M.; Tankó, L.B.; Schubert-Tennigkeit, A.A.; et al. Efficacy and Safety of Bimagrumab in Sporadic Inclusion Body Myositis: Long-Term Extension of RESILIENT. *Neurology* **2021**, doi:10.1212/WNL.0000000000011626.
97. Rooks, D.; Praestgaard, J.; Hariry, S.; Laurent, D.; Petricoul, O.; Perry, R.G.; Lach-Trifilieff, E.; Roubenoff, R. Treatment of Sarcopenia with Bimagrumab: Results from a Phase II, Randomized, Controlled, Proof-of-Concept Study. *J. Am. Geriatr. Soc.* **2017**, *65*, 1988–1995, doi:10.1111/jgs.14927.
98. Rooks, D.; Swan, T.; Goswami, B.; Filosa, L.A.; Bunte, O.; Panchaud, N.; Coleman, L.A.; Miller, R.R.; Garcia Garayoa, E.; Praestgaard, J.; et al. Bimagrumab vs Optimized Standard of Care for Treatment of Sarcopenia in Community-Dwelling Older Adults: A Randomized Clinical Trial. *JAMA Netw. Open* **2020**, *3*, e2020836, doi:10.1001/jamanetworkopen.2020.20836.
99. Rooks, D.S.; Laurent, D.; Praestgaard, J.; Rasmussen, S.; Bartlett, M.; Tankó, L.B. Effect of Bimagrumab on Thigh Muscle Volume and Composition in Men with Casting-induced Atrophy. *J. Cachexia Sarcopenia Muscle* **2017**, *8*, 727–734, doi:10.1002/jcsm.12205.
100. Polkey, M.I.; Praestgaard, J.; Berwick, A.; Franssen, F.M.E.; Singh, D.; Steiner, M.C.; Casaburi, R.; Tillmann, H.-C.; Lach-Trifilieff, E.; Roubenoff, R.; et al. Activin Type II Receptor Blockade for Treatment of Muscle Depletion in Chronic Obstructive Pulmonary Disease. A Randomized Trial. *Am. J. Respir. Crit. Care Med.* **2019**, *199*, 313–320, doi:10.1164/rccm.201802-0286OC.
101. Mendell, J.R.; Sahenk, Z.; Malik, V.; Gomez, A.M.; Flanigan, K.M.; Lowes, L.P.; Alfano, L.N.; Berry, K.; Meadows, E.; Lewis, S.; et al. A Phase 1/2a Follistatin Gene Therapy Trial for Becker Muscular Dystrophy. *Mol. Ther.* **2015**, *23*, 192–201, doi:10.1038/mt.2014.200.
102. Mendell, J.R.; Sahenk, Z.; Al-Zaidy, S.; Rodino-Klapac, L.R.; Lowes, L.P.; Alfano, L.N.; Berry, K.; Miller, N.; Yalvac, M.; Dvorchik, I.; et al. Follistatin Gene Therapy for Sporadic Inclusion Body Myositis Improves Functional Outcomes. *Mol. Ther. J. Am. Soc. Gene Ther.* **2017**, *25*, 870–879, doi:10.1016/j.ymthe.2017.02.015.
103. Padhi, D.; Higano, C.S.; Shore, N.D.; Sieber, P.; Rasmussen, E.; Smith, M.R. Pharmacological Inhibition of Myostatin and Changes in Lean Body Mass and Lower Extremity Muscle Size in Patients Receiving Androgen Deprivation Therapy for Prostate Cancer. *J. Clin. Endocrinol. Metab.* **2014**, *99*, E1967–E1975, doi:10.1210/jc.2014-1271.

.
